# An efficient strategy for producing a stable, replaceable, highly efficient transgene expression system in silkworm, *Bombyx mori*

**DOI:** 10.1038/srep08802

**Published:** 2015-03-05

**Authors:** Dingpei Long, Weijian Lu, Yuli Zhang, Lihui Bi, Zhonghuai Xiang, Aichun Zhao

**Affiliations:** 1 State Key Laboratory of Silkworm Genome Biology, Key Laboratory for Sericulture Functional Genomics and Biotechnology of Agricultural Ministry, Southwest University, Chongqing 400716, People's Republic of China; 2 Guangxi Research Academy of Seficultural Science, Guangxi Nanning 530007, People's Republic of China

## Abstract

We developed an efficient strategy that combines a method for the post-integration elimination of all transposon sequences, a site-specific recombination system, and an optimized fibroin H-chain expression system to produce a stable, replaceable, highly efficient transgene expression system in the silkworm (*Bombyx mori*) that overcomes the disadvantages of random insertion and post-integration instability of transposons. Here, we generated four different transgenic silkworm strains, and of one the transgenic strains, designated TS1-RgG2, with up to 16% (w/w) of the target protein in the cocoons, was selected. The subsequent elimination of all the transposon sequences from TS1-RgG2 was completed by the heat-shock-induced expression of the transposase *in vivo*. The resulting transgenic silkworm strain was designated TS3-g2 and contained only the *attP*-flanked optimized fibroin H-chain expression cassette in its genome. A phiC31/*att*-system-based recombinase-mediated cassette exchange (RMCE) method could be used to integrate other genes of interest into the same genome locus between the *attP* sites in TS3-g2. Controlling for position effects with phiC31-mediated RMCE will also allow the optimization of exogenous protein expression and fine gene function analyses in the silkworm. The strategy developed here is also applicable to other lepidopteran insects, to improve the ecological safety of transgenic strains in biocontrol programs.

Since the first report of insect transformation with the *P* element in *Drosophila melanogaster*^1^, transposon-based germline transformation systems had been widely used for both basic and applied studies in insects and other invertebrates over the past 30 years^2^,^3^. At present, four transposon vector systems have been used relatively extensively for the transformation of insect species. These include the *mariner* family transposon *Mos1* isolated from *D. mauritiana*^4^ and *Minos* isolated from *D. hydei*^5^, the *hAT*-related transposon *Hermes* isolated from *Musca domestica*^6^, and the *TTAA*-specific family transposon *piggyBac* derived from *Trichoplusia ni*^7^. *piggyBac* is a distinct class II transposon, encoding a 594-amino-acid transposase that mediates its excision and reinsertion^8^. The transposition of *piggyBac* occurs in a precise cut-and-paste fashion and targets TTAA sites in the host genome^7^,^9^. *piggyBac* is currently the most widely used transposon vector and has been successfully used for transgenic engineering in more than 30 insect species, including *D. melanogaster*^10^, *Ceratitis capitata*^11^, *Tribolium castaneum*^12^, *Bombyx mori*^13^, *Aedes aegypti*^14^, and *Apis mellifera*^15^. It facilitates the extension of enhancer-trapping strategies, allowing the identification and functional analysis of genes, the expression of foreign proteins, the biological control of pest species, and other genetic engineering operations^3^.

By far the most frequently published application of nondrosophilid insect transgenesis has been the transformation of the silkworm, *B. mori*, using *piggyBac*^3^. This useful model lepidopteran and economically important insect has been used as an effective protein bioreactor for more than 5000 years in the production of silk^16^. At present, the silk glands of silkworms have been developed as fine bioreactors for the production of recombinant proteins from transgenic expression vectors, all based on *piggyBac*-mediated germline transformation, using the various promoters of the sericin 1 (*Ser1*), fibroin heavy chain (H-chain; *FibH*), fibroin light chain (L-chain; *FibL*), and fibrohexamarin genes (*fhx*)^17^,^18^,^19^. We previously constructed an optimized fibroin H-chain expression system to express a recombinant fusion protein in the silk glands of silkworms, and the recombinant fusion protein constituted up to 15% (w/w) of the transgenic silkworm cocoon^20^. To our knowledge, this is still the most efficient silkworm silk gland expression system to date. Methods using *piggyBac* to introduce exogenous DNA into the silkworm genome are characterized by random integration^13^, which could be used to screen for favorable insertion loci for the highly efficient expression of exogenous proteins in transgenic silkworms. It would thus contribute to the utilization of silkworm bioreactors for the commercial production of exogenous proteins^19^. However, position effects and insertional mutagenesis during *piggyBac*-mediated random integration can have several side effects, including unpredictable variations in gene expression, disruption of the gene structure of the host, and the reduced fitness of the transgenic strain^21^. It is also almost impossible to repeatedly introduce several different exogenous genes into a specific target locus using only *piggyBac* or other transposons.

Site-specific recombinase (SSR) systems have been developed using recombinases that catalyze the exchange of DNA strands between two target recombination sites, in contrast to the random insertion of genes by transposons^22^. SSR systems provide many advantages for transgenic engineering that are lacking in transposon-based systems. The capacity for reproducible insertion into genetically active loci can be useful in defining and utilizing chromosomal sites with low silencing potential^3^. Currently, the most commonly used SSR systems are FLP/*FRT* from the 2 μm plasmid of *Saccharomyces cerevisiae*^23^, Cre/*loxP* from *Escherichia coli* phage P1^24^, and phiC31/*att* from the *Streptomyces* phage phiC31^25^. These systems have been successfully used for the site-specific integration of transgenes in the genomes of mosquitoes, *D. melanogaster*, and the fruit fly *C. capitata*^3^. Recently, the recombination activity of these three recombinases has also been used to manipulate the silkworm genome^26^,^27^,^28^. Duan et al. demonstrated that the Cre/*loxP* system can be used to control the activation and expression of marker genes in the middle silk gland cells of transgenic silkworms^27^. We previously used the FLP/*FRT* system to site-specifically excise a target gene at predefined chromosomal sites in the silkworm^26^. We also used the phiC31/*att* system to produce heritable site-specific transgene integration into predetermined chromosomal sites in the transgenic silkworm genome with phiC31-mediated cassette exchange (phiC31-mediated recombinase-mediated cassette exchange [RMCE]) reaction^28^. Although these SSR systems have been developed as effective targeted recombination systems in a variety of insect species, they have one drawback: naturally occurring integrase sites are extremely rare in the genomes of insects^3^. Occasionally, there may be functional pseudointegration sites, but these sites are generally not ideal and cannot be targeted by integrases with the frequency of native recombination sites^3^. Therefore, it is necessary in many insect species, including *D. melanogaster*^29^,^30^, *C. capitata*^31^, mosquitoes^32^,^33^,^34^, and silkworms^28^,^35^, to first introduce a canonical site (such as *FRT*, *loxP*, *attP*, or *attB*) into their genomes using transposon-mediated germline transformation.

One potential concern relating to the field use of transgenic insects is that using transposons as gene vectors may lead to postinsertion instability of the transgene. It has been reported that integrated *piggyBac* can be remobilized in the genomes of *D. melanogaster*^36^, *C. capitata*^37^, *Anastrepha ludens*^38^, *T. castaneum*^39^, *Anopheles stephensi*^40^, *Harmonia axyridis*^41^, and *B. mori*^42^. We have also observed the phenomenon of *piggyBac* remobilization during the large-scale rearing of commercial strains of transgenic silkworms^43^. This phenomenon can be caused by the unintended presence of a mobilizing transposase, which may have been undetected in endogenous *piggyBac*-like transposons or subsequently entered the host species by horizontal gene transfer^21^. *Bombyx mori* is a domesticated organism, completely dependent on humans for its survival and reproduction^16^, so its exposure to exogenous transposases by horizontal gene transfer is unlikely. However, according to some previous reports, at least 100 *piggyBac*-like sequences (BmPBLE1–98, yabusame-1, and yabusame-W) are present in the silkworm genome, and some of these transposons might encode potential transposase activity^44^,^45^. Therefore, endogenous *piggyBac*-like transposase activity cannot be avoided in the silkworm. In contrast, the *piggyBac* transpose *via* an internally encoded transposase acting on the flanking 5′ and 3′ terminal inverted repeat (TIR) sequences and adjacent DNA, which may include subterminal inverted repeat sequences^46^. In principle, *piggyBac*-based vectors can be stabilized by the deletion of one or both TIRs after their genomic integration. Indeed, all *piggyBac*-derived sequences can be eliminated with this method, including the selective marker genes used for the initial germline transformation. Until now, this method has been successfully used for the post-integration stabilization of transgenes in the genomes of *D. melanogaster*^47^, *C. capitata*^37^, *Anastrepha ludens*^38^, and more recently, *H. axyridis*^41^. However, this strategy has not yet to be used in *B. mori* or any other lepidopteran species. To overcome the disadvantages of the position effects and potential insertional mutagenesis incurred by *piggyBac*-mediated random integration and to provide a stable, replaceable, and highly efficient expression system, a combination of the *piggyBac*- and SSR-based systems was developed, which had not previously been established in *B. mori* or any other species *in vivo*.

Based on the considerations discussed above, we developed an efficient strategy for producing a stable, replaceable, and highly efficient transgene expression system in the silkworm. First, we used *piggyBac*-mediated germline transformation to generate a transgenic silkworm strain that produces exogenous proteins with high efficiency in the silk gland and characterized the strain. The subsequent elimination of all transposon sequences, including the marker genes used for the initial germline transformation, resulted in the post-integration stabilization of the target gene expression cassette of interest in the transgenic silkworm genome. Because this expression cassette was flanked by two *attP* sites, a phiC31-mediated RMCE system can be used to repetitively integrate other gene expression cassettes into the same genomic locus. Our strategy offers a novel way to establish stable and replaceable transgenic silkworm strains for use as protein bioreactors and for fine gene function analyses. It will facilitate the development of lepidopteran species carrying stabilized transgenic insertions for both basic and applied purposes, including the comparative analysis of true transgenic alleles and the biological control of pest species. Our study also provides insight into the further improvement of various genetic manipulation systems.

## Results

### Plasmid and experimental design

**Figure 1:**
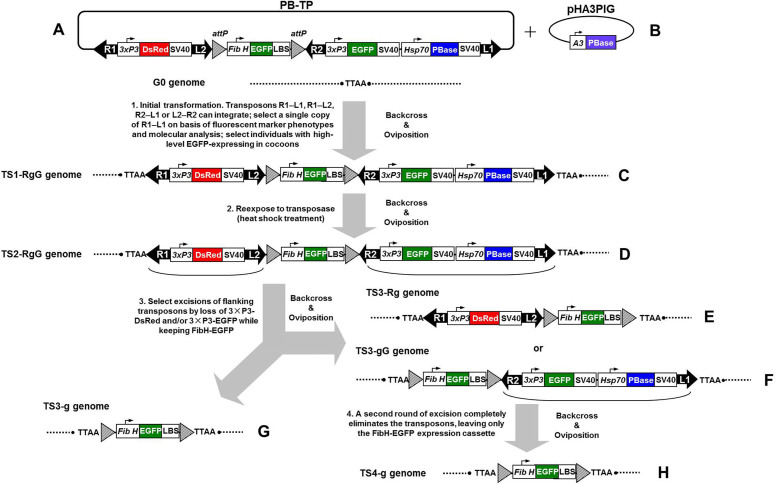
Strategy for the selection of high-efficiency transgene expression and the post-integration stabilization of the transgene using PB-TP vector in silkworms. The initial transformation uses the PB-TP vector (A) carrying a composite transposon comprising two pairs of opposed transposon arms. The PB-TP structurally combines four different transposons (R1–L1, R1–L2, R2–L1, and L2–R2). The desired transposon R1–L1 was inserted into the TTAA site of the G0 silkworm germ cell genome to produce the TS1-RgG individuals (C) mediated by *piggyBac* transposase derived from plasmid pHA3PIG (B). TS1-RgG individuals containing a single copy of the R1–L1 construct in their genomes (selected by the 3×P3-DsRed, FibH-EGFP and 3×P3-EGFP markers) and expressing high-level EGFP in their cocoons were identified. The flanking transposons (R1–L2 and R2–L1) can then be eliminated in TS2-RgG germ-cell genome (D) by reexposure to transposase, mediated by heat shock treatment, resulting in the removal of one flanking transposon (R2–L1 or R1–L2) or both flanking transposons from the TS3-Rg, TS3-gG, or TS3-g genome (E–G). TS3-gG individuals in which only the R1–L2 transposon was deleted from the genome (F) were also used for a secend round of excision, completely eliminating the transposons (as above), leaving only the FibH-EGFP expression cassette flanked by two 39-bp *attP* sites (*triangles with diagonal lines*) in the same orientation in the TS4-g genome (H). 3×P3, 3×P3 promoter; DsRed, *DsRed* gene (*red box*); SV40, SV40 polyadenylation signal sequence; FibH, 5′-flanking sequence of the silkworm fibroin heavy chain gene (~2.3-kb); EGFP, *EGFP* gene (*green box*); LBS, light chain binding site sequence of the silkworm fibroin heavy chain gene; Hsp70, *D. Melanogaster* *hsp70* promoter; A3, silkworm cytoplasmic actin 3 promoter; PBase, *piggyBac* transposase gene (*blue box*).

Figure 1A shows the structure of the *piggyBac*-derived target plasmid (PB-TP) vector. Details of the construction procedure are described in the Methods section. The PB-TP vector containing an FibH-EGFP-LBS expression cassette (FibH, 5′-flanking sequence of *FibH* gene; EGFP, enhanced green fluorescent protein; LBS, L-chain binding site) was placed between two phiC31 integrase recognition sites (*attP*), and flanked by two short *piggyBac* arms L2 and R2; a 3×P3-promoter-driven *DsRed* gene expression cassette, 3×P3-DsRed-SV40 (SV40, SV40 polyadenylation signal sequence [polyA]), was placed between *piggyBac* arms R1 and L2, and a 3×P3-promoter-driven *EGFP* gene expression cassette, 3×P3-EGFP-SV40, was placed between *piggyBac* arms R2 and L1; a *Drosophila* heat shock protein 70 (*hsp70*)-promoter-driven *piggyBac* transposase (PBase) gene expression cassette, Hsp70-PBase-SV40, which was used to express PBase *in vivo* with heat shock treatment (HST), was placed behind the 3×P3-EGFP-SV40 expression cassette and also between R2 and L1. Thus, PB-TP structurally combines four different transposons (R1–L1, R1–L2, R2–L1, and L2–R2) that can potentially be expressed from this type of construct, and each transposon can be identified by a different combination of the 3×P3-DsRed (R), 3×P3-EGFP (G), and FibH-EGFP (g) fluorescent markers (Figure 1A). Supplementary Figure S1 shows the constructs and the sequences of the *piggyBac* arms used in this study.

As described in the Methods section of this study, “G1 transgenic strain (TS)” is abbreviated as “TS1”, and subsequent TS generations are thus referred to as TS2, TS3, etc. TSn individuals with different fluorescent phenotypes, such as 3×P3-DsRed (R), FibH-EGFP (g), or 3×P3-EGFP (G), are abbreviated to TSn-R, TSn-G, or TSn-g, respectively (n = generations). For example, the transgenic individuals from G1 generations displaying three kinds of fluorescence were designated “TS1-RgG”. To select for high-efficiency transgene expression and the post-integration stability of the transgene by eliminating all the transposon sequences from the silkworm, we proceeded as follows steps (illustrated in Figure 1). (*i*) The PB-TP vector was integrated into the *B. mori* 871 strain with the *piggyBac*-mediated germline transformation of diapause silkworm strains^48^, and TS1-RgG individuals containing a single copy of the transposon R1–L1 construct in their genomes and expressing high-level EGFP in their cocoons were identified by their fluorescent marker phenotypes and with a molecular analysis (Figure 1C). (*ii*) Nondiapause heterozygous TS2-RgG individuals were treated with HST in the embryonic or larval stage. Remobilization of the flanking transposons (R1–L2 and R2–L1) in the TS2-RgG germ-cell genome was mediated by PBase expressed with HST (Figure 1D), resulting in the removal of one flanking transposon (R2–L1 or R1–L2) or both flanking transposons from the TS3-Rg, TS3-gG, or TS3-g genome (Figure 1E–G). (*iii*) Remobilized TS3 individuals were identified by their fluorescent marker phenotypes. (*iv*) TS3-gG individuals in which only the R1–L2 transposon was deleted from the genome (Figure 1F) were also used for a second round of excision, completely eliminating the transposons (as above), leaving only the FibH-EGFP expression cassette flanked by two 39-bp *attP* sites in the same orientation in the TS4-g genome (Figure 1H). This method not only induces post-integration stabilization in the transgenic silkworm described above, but also allows precise cassette replacement with cassettes containing different genes of interest *via* a phiC31-mediated RMCE reaction.

### Screening TS1-RgG for high-efficiency transgene expression

As shown in Table 1, different numbers of DsRed- and/or GFP-positive G1 broods and larvae were obtained from two independent injection experiments, as described in the Methods. In total, we obtained 11 G1 transgenic-positive broods, each containing at least one transgenic larva (designated the “positive brood”). The percentages of positive G1 broods produced from two independent injection experiments were 13.6% and 10.3% (Table 1). Because the PB-TP vector encodes four potential transposons, as described above, the TS1 larvae display different fluorescence phenotypes with the insertion of different transposons. The analysis of the different fluorescence phenotypes of the TS1 individuals from the positive G1 broods is shown in Table 2. Supplementary Figure S2 shows fluorescent images of TS1 silkworms in the early larval stage. It is noteworthy that the four screened TS1-RG larvae from one positive G1 brood (Table 2) not only had the L2–R2 insertion, but also the simultaneous insertion of both R1–L2 and R2–L1 into the genomes of TS1 individuals (Supplementary Figure S2D). However, as described in Supplementary Figure S3, the polymerase chain reaction (PCR) results confirmed that each TS1-RG individual in this positive G1 brood contained only the L2–R2 construct in its genome, and the simultaneous insertion of both R1–L2 and R2–L1 was not detected.

**Figure 2:**
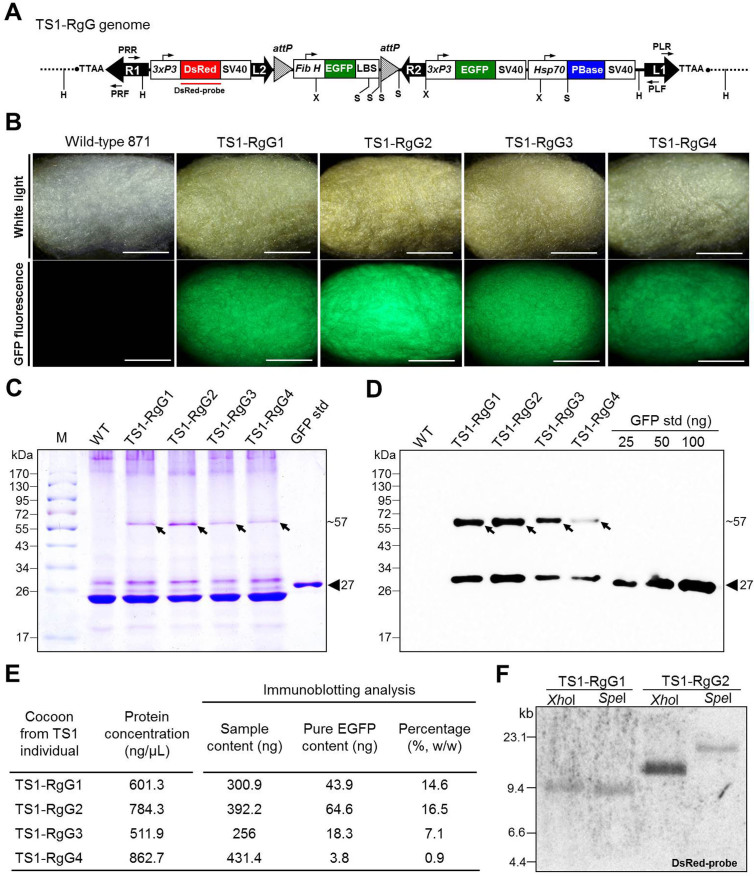
Analysis of the expression of recombinant EGFP in the cocoons from TS1-RgG silkworms and insertion copy number of R1–L1 transgene construct in TS1-RgG silkworms. (A) Schematic map of the R1–L1 transgene construct in genome of TS1-RgG individual. PLF/PLR and PRF/PRR are the *piggyBac* left arm 1 (L1) and right arm 1 (R1) primer pairs, respectively. H, *Hae*III; X, *Xho*I; S, *Spe*I. (B) The cocoons from wild-type 871 and TS1-RgG1–TS1-RgG4 silkworms were observed under white light (exposure time 1/800 sec) and GFP-fluorescence (exposure time 1/10 sec). White scale bar represents 1 cm. (C) Cocoon silk protein samples from wild-type 871 (WT), TS1-RgG1–TS1-RgG4 silkworms were subjected to SDS-12% PAGE with 2-ME treatment. Arrowheads indicate recombiant EGFP (~57-kDa), and triangle indicates the GFP standard (27-kDa). M is molecular mass markers. Sizes are indicated on the left of the panels. (D) Immunoblotting analysis of proteins in the gel as in (C) with an anti-GFP antibody. Arrowheads indicate recombiant EGFP, triangle indicates the GFP standard (25 ng, 50 ng and 100 ng). Sizes are indicated on the left of the panels. (E) Comparison of the content of recombinant proteins in the cocoons from different TS1-RgG individuals. The ratio of pure EGFPs to the cocoon silk proteins within the membrane lanes were quantified by densitometry with Image-J software and the GFP standard used as a control. (F) Southern blotting analysis of XhoI- or SpeI-digested genomic DNA using DsRed probe. The individual DNA hybridization patterns of the XhoI- or SpeI-digested TS1-RgG1 and TS1-RgG2 lanes are shown. Sizes are indicated on the left of panel.

As shown in Table 2, four TS1-RgG individuals were obtained from four of the 11 positive G1 broods for subsequent experiments and designated TS1-RgG1–TS1-RgG4. Because all the TS1-RgG individuals contained the FibH-EGFP-LBS expression cassette in their genomes (Figure 2A), the cocoons from the TS1-RgG1–TS1-RgG4 silkworms displayed strong green fluorescence, indicating a large amount of recombinant EGFP was spun into their cocoons (Figure 2B). The cocoon from TS1-RgG2 displayed the strongest fluorescence among the four cocoons at the same exposure time and excitation light intensity (Figure 2B). The cocoon silk proteins from the TS1-RgG and wild-type 871 silkworms were analyzed with SDS-PAGE and immunoblotting with an anti-GFP antibody (Figure 2C and D). The concentrations of the cocoon proteins extracted from the different TS1-RgG individuals ranged from 511.9 to 862.7 ng/μL (Figure 2E). The results of SDS-PAGE suggested that the EGFP/H-chain fusion proteins derived from each TS1-RgG individual were single proteins of about 57 kDa (Figure 2C, arrowhead). Based on the results of immunoblotting (Figure 2D), we calculated that the contents of pure EGFP in the TS1-RgG1–TS1-RgG4 silkworm cocoons were 14.6%, 16.5%, 7.1%, and 0.9% (w/w), respectively (Figure 2E), which is consistent with the fluorescence stereomicroscopic observations.

Southern blotting and inverse PCR were used to determine the copy numbers and insertion positions of the transgene construct in the TS1-RgG1 and TS1-RgG2 individuals (Figure 2A). Southern blotting showed that both the TS1-RgG1 and TS1-RgG2 adults contained only one copy of the R1–L1 transgene construct (Figure 2F). The 20-bp silkworm genomic sequences flanking the *piggyBac* arms are shown in Table 3. Both TS1-RgG1 and TS1-RgG2 carried the transgene in a heterozygous state. The R1–L1 inserts in the genomes of TS1-RgG1 and TS1-RgG2 were located on chromosomes 24 and 18, respectively. Thus, we had established two heterozygous G1 TSs, TS1-RgG1 and TS1-RgG2, containing a single copy of the R1–L1 transgene construct in their genomes. Because the TS1-RgG2 individual more efficiently expressed the recombinant EGFP protein in its cocoon, it was selected for subsequent experiments.

### Production of transposon-free and marker-free transgenic silkworms

**Figure 3:**
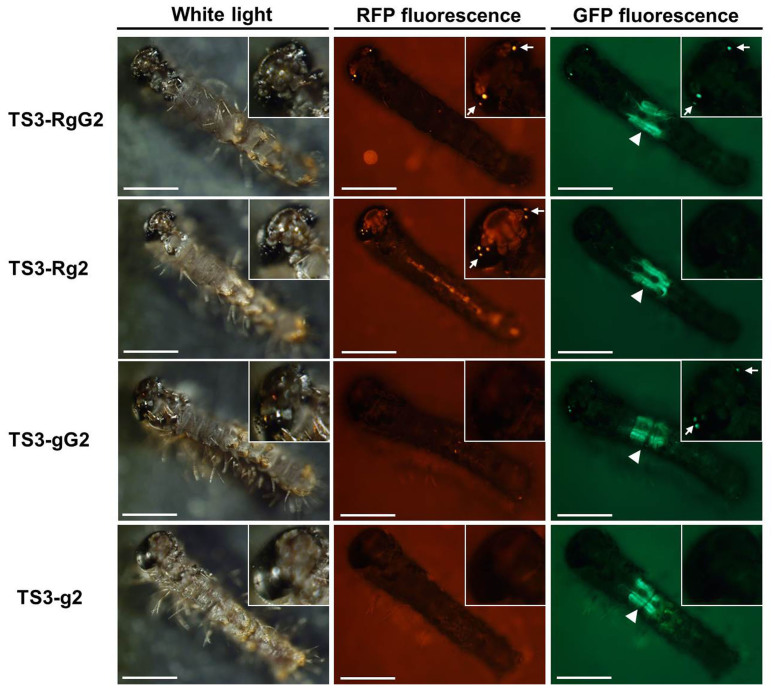
Expression of DsRed and EGFP genes in the larvae of TS3 silkworms. The newly hatched larvae of TS3-RgG2, TS3-Rg2, TS3-gG2 and TS3-g2 were observed under white light, RFP-fluorescence and GFP-fluorescence. Arrowheads denote the position of the RFP or/and GFP fluorescence in the larval ocelli. Triangles denote the position of the GFP fluorescence in the larval silk glands. White scale bar represents 1 mm.

As described in the Methods section and illustrated in Supplementary Figure S4A, one heterozygous TS1-RgG2 adult (male ♂) was backcrossed with a wild-type 871 adult (female ♀) to produce G2 broods. The individuals in different groups from the same G2 brood were treated with HST in the embryonic stage (group 2^#^), in the larval stage (group 3^#^), or without HST (group 1^#^) (Supplementary Figure S4B). The results of screening the TS2 individuals from each group are shown in Table 4. The screened G3 broods containing at least one TS3-Rg2, TS3-gG2, or TS3-g2 larva were designated the Rg-, gG-, or g-positive broods, respectively. Finally, we obtained six g-positive broods from the 37 G3 broods from group 2^#^ and one g-positive brood from the 45 G3 broods from group 3^#^ that had at least one TS3-g2 larva. The frequencies of g-positive broods in the G3 broods of groups 2^#^ and 3^#^ were 16.22% (6/37) and 2.22% (1/45), respectively (Table 5). The frequencies of G3 broods containing at least one TS3-Rg2, TS3-gG2, or TS3-g2 larva in the G3 broods of groups 2^#^ and 3^#^ were 62.16% (27/37) and 15.56% (7/45), respectively (Table 5). Although there was no g-positive brood in any of the G3 broods from group 1^#^, two gG-positive broods from the 48 G3 broods from group 1^#^ were identified (Table 5). This result implies the background expression of PBase from the *hsp70* promoter in the control, which would also lead to the remobilization event in the silkworm. Figure 3 shows the expression of the DsRed and EGFP genes in the larvae of TS3 individuals.

**Figure 4:**
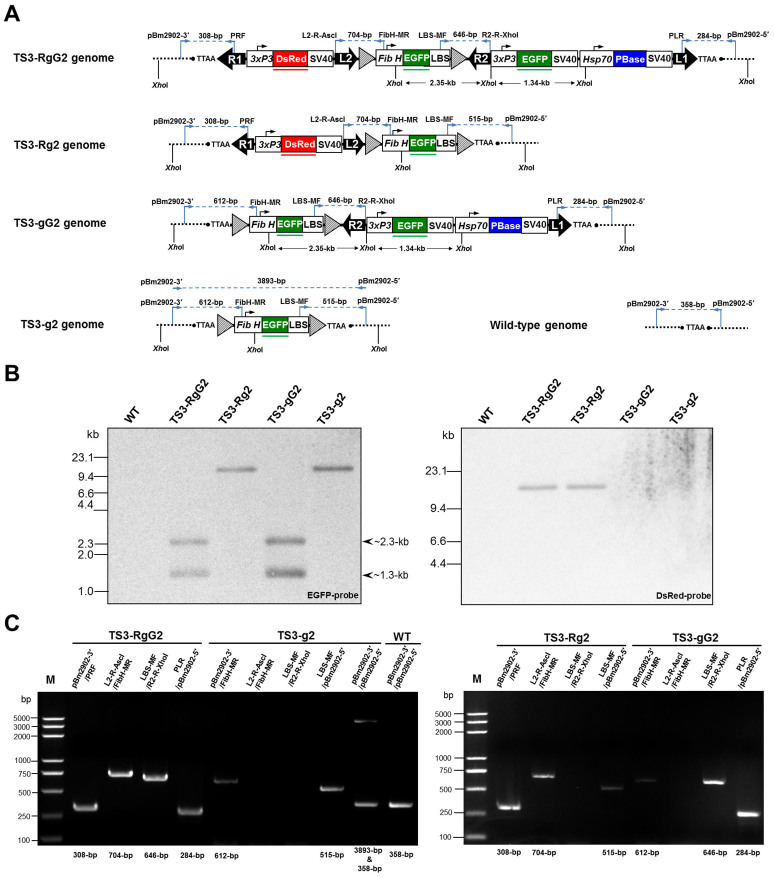
Molecular confirmation of the deletion of the flanking transposons in the TS3 individuals by *piggyBac* remobilization. (A) Schematic of four different transgene constructs in genomes of TS3 silkworms and the same genome site of wild-type 871 silkworms. The relative primer positions were indicated above the diagram of different transgene constructs, and the suitable primer pairs were used for PCR analysis of genomic DNA. Primers pBm2902-3′ and pBm2902-5′ were each designed from the 3′ and 5′ *piggyBac*-flanking genomic sequences of TS1-RgG2 genome based on the results of inverse PCR. Primers FibH-MR and LBS-MF were each designed from the H-chain gene promoter and L-chain binding site sequencs of the *B. mori* fibroin H-chain gene (Supplementary Table S1). Red and green lines indicate the region homologous to the DsRed probe and EGFP probe, respectively. The XhoI restriction enzyme sites for the transgene constructs are shown. (B) Southern blotting analysis of XhoI-digested genomic DNA using EGFP probe (*left*) and DsRed probe (*right*), respectively. The individual DNA hybridization patterns of the wild-type 871 (WT), TS3-RgG2, TS3-Rg2, TS3-gG2 and TS3-g2 lanes are shown. Sizes are indicated on the left of panel. (C) PCR confirmation of genomic DNA using suitable primer pairs. The left panel shows the PCR products of genomic DNA from individuals of TS3-RgG2, TS3-g2 and wild-type 871 (WT), and the right panel shows the PCR products of genomic DNA from individuals of TS3-Rg2 and TS3-gG2. Lane M, Trans2K Plus DNA Marker. Sizes of the PCR products are shown below each lane.

To confirm the deletion of the flanking transposons (R1–L2 and/or R2–L1) in the TS3 individuals by *piggyBac* remobilization, a Southern blotting analysis was performed on XhoI-digested genomic DNA from different TS3 adults and wild-type 871 adults using the EGFP and DsRed probes, respectively (Figure 4A). As shown in Figure 4B, the EGFP probe hybridizing with the *EGFP* gene recognized 2.3-kb and 1.3-kb fragments in samples from TS3-RgG2 and TS3-gG2 individuals, respectively, whereas the samples from TS3-Rg2 and TS3-g2 individuals showed only one band of the same size when blotted with the EGFP probe, and there was no hybridization in the sample from a wild-type 871 individual. The DsRed probe hybridizing with the *DsRed* gene recognized only one fragment of the same size in the samples from TS3-RgG2 and TS3-Rg2 individuals, and did not hybridize with the samples from the TS3-gG2, TS3-g2, and wild-type 871 individuals. These results are consistent with the expected pattern.

The transgene structures in the TS3 individuals were also confirmed with a PCR analysis using primers complementary to the genomic DNA and internal vector DNA (Supplementary Table S1), which yielded product sizes consistent with the deletion of R2–L1 from the TS3-Rg2 individuals, the deletion of R1–L2 from the TS3-gG2 individuals, and the deletion of both R2–L1 and R1–L2 from the TS3-g2 individuals (Figure 4A and C). Because the TS3-g2 individuals were heterozygous for the transgene insertion, the PCR products were a 3893-bp DNA fragment that spanned the *attP*-flanked FibH-EGFP-LBS expression cassette sequence and a 358-bp DNA fragment from the wild-type *B. mori* genome for the TS3-g2 individuals when the primer pair pBm2902-3′/pBm2902-5′ was used. The PCR product from the wild-type 871 individuals was only a 358-bp DNA fragment when the same primer pair was used. The PCR products from all TS3-g2 individuals were sequenced, and no structural changes were detected in either the cassette itself or the TS3-g2 genomic DNA, indicating that *piggyBac* was excised without leaving a footprint at the excision-site TTAA element, as we expected. Supplementary Figure S5 shows the sequencing results for the 3′ and 5′ sequences of the *attP*-flanked FibH-EGFP-LBS expression cassette in the genomes of the TS3-g2 individuals and for the wild-type genomic sequence at the same site in the wild-type 871 individuals.

A few TS2-R2 and TS2-gG2 individuals were also screened from each group of this G2 brood (Table 4). The inverse PCR results confirmed that the R1–L2 transgene construct was located at an identical site on chromosome 6 in the genomes of all the TS2-R2 individuals, and the transgene construct in the genomes of the TS2-RgG2 and TS2-gG2 individuals was located on chromosome 18, as in the TS1-RgG2 male (Supplementary Table S2). These results suggest that the R1–L2 remobilization event occurred in the spermatocytes of the TS1-RgG2 male, and also confirm the background expression activity of the *hsp70* promoter in the silkworm.

### Characterization of the optimal HST strategy for producing transposon-free transgenic silkworms

**Figure 5:**
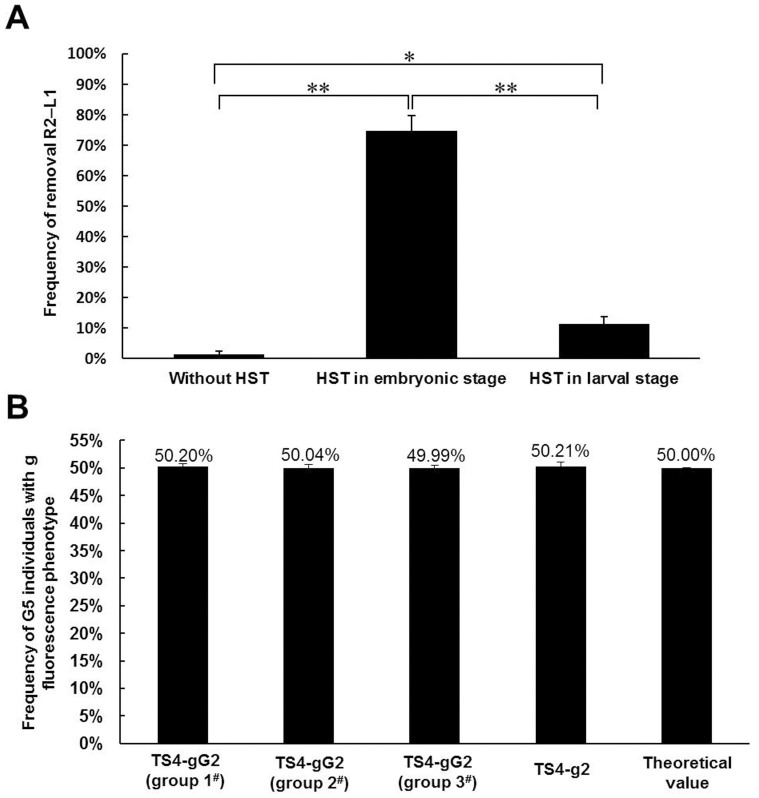
Analysis of the removal of R2–L1 in the TS3-gG2 offspring and the stability of g fluorescence phenotype in the TS3-gG2 and TS3-g2 offspring. (A) Comparison of the frequency of the removal R2–L1 by HST or without HST in TS3-gG2 offspring using the data from Supplementary Table S3. Bars represent the standard deviations (*n* = 3). Statistically significant differences: *P < 0.01, **P < 0.001. (B) Comparison of the rates of G5 individuals with g fluorescence phenotype from each of the G5 broods obtained by crossing heterozygous TS4-gG2 or heterozygous TS4-g2 adults with wild-type 871 adults. According to the data from Supplementary Table S3 and our original data, the rates of G5 individuals with g fluorescence phenotype (including TS5-gG2 and TS5-g2 individuals) from 150 G5 broods of each experimental group obtained by crossing heterozygous TS4-gG2 adults with wild-type adults were 50.2% (group 1^#^, 37872/75446), 50.04% (group 2^#^, 37595/75127), and 49.99% (group 3^#^, 37698/75410), respectively. According to the data from Supplementary Table S4, the rate of G5 individuals with g fluorescence phenotype (TS5-g2 individuals) from 8 G5 broods obtained by crossing heterozygous TS4-g2 adults with wild-type 871 adults was 50.21% (2049/4081). The theoretical value of G5 individuals with g fluorescence phenotype from each of the G5 broods should be 50%. Bars represent the standard deviations. There were no statistically significant differences in the rates of G5 individuals with g fluorescence phenotype among all experiments for the statistical evaluation of FibH-EGFP expression cassette stability (*P* > 0.05).

To identify the optimal HST strategy for producing transposon-free transgenic silkworms, as illustrated and described in Figure S6, one heterozygous TS3-gG2 male was backcrossed with three different wild-type 871 females (a, b, and c) to produce three G4 broods, and the G4 individuals from the three groups (1^#^, 2^#^, and 3^#^) of each G4 brood were treated with or without HST, as described in Supplementary Figure S4B. Finally, the fluorescent phenotypes of the larvae from 50 G5 broods of each group (G5 a, b and c broods) were analyzed (Supplementary Figure S6). The results suggest that the frequency of g-positive broods in the G5 broods of group 1^#^ (without HST), group 2^#^ (HST in the embryonic stage), and group 3*^#^* (HST in the larval stage) were 0%–2%, 70%–80%, and 10%–14%, respectively (Supplementary Table S3). Thus, the frequency of the removal of R2–L1 by HST was significantly higher in the embryonic stage than in the larval stage or without HST, and the frequency of the removal of R2–L1 by HST was also significantly higher in the larval stage than without HST (Figure 5A).

### Detection of genetic stability of the FibH-EGFP expression cassette in TS3 offspring

To estimate the stability of the FibH-EGFP expression cassette in the offspring of TS3-g2, 16 G5 broods were obtained by the reciprocal crosses between TS4-g2 (+/−, indicates a heterozygote) or TS4-g2 (+/+, indicates a homozygote) adults and wild-type 871 adults, and the fluorescent phenotypes of the larvae from these G5 broods were analyzed. As shown in Supplementary Table S4, the rate of TS5-g2 individuals from each of the 8 G5 broods (total 4081 G5 individuals) obtained by the reciprocal crosses between TS4-g2 (+/−) adults and wild-type 871 adults were almost exactly 50% (if the g is stable, the theoretical rate of g phenotype in these G5 broods should be 50%), and the rate of TS5-g2 individuals from each of the 8 G5 broods (total 4059 G5 individuals) obtained by the reciprocal crosses between TS4-g2 (+/+) adults and wild-type 871 adults were all 100% (if the g is stable, the theoretical rate of g phenotype in these G5 broods should be 100%). The results confirmed the stability of the FibH-EGFP expression cassette in the absence of PBase in the TS3-g2 offspring.

As illustrated and described in Figure S6 and shown in Supplementary Table S3, all the TS4-gG2 adults were heterozygous for the FibH-EGFP expression cassette insertion. These results suggested that the rates of TS5-g2 individuals from each of the G5 g-positive broods were 1.53%–25.78%, which indicated the frequencies of R2–L1 remobilization in each of the G5 g-positive broods were at least 1.53%–25.78%. But the rates of G5 individuals with g fluorescence phenotype (including TS5-gG2 and TS5-g2 individuals) from 150 G5 broods of each experimental group (total more than 75000 G5 individuals of each experimental group) were also almost exactly 50% (Figure 5B and Supplementary Table S3), which was consistent with the theoretical value. The results confirmed that the FibH-EGFP expression cassette cannot be remobilized in the genomes of TS4-gG2 individuals when PBase is present *in vivo*.

**Figure 6:**
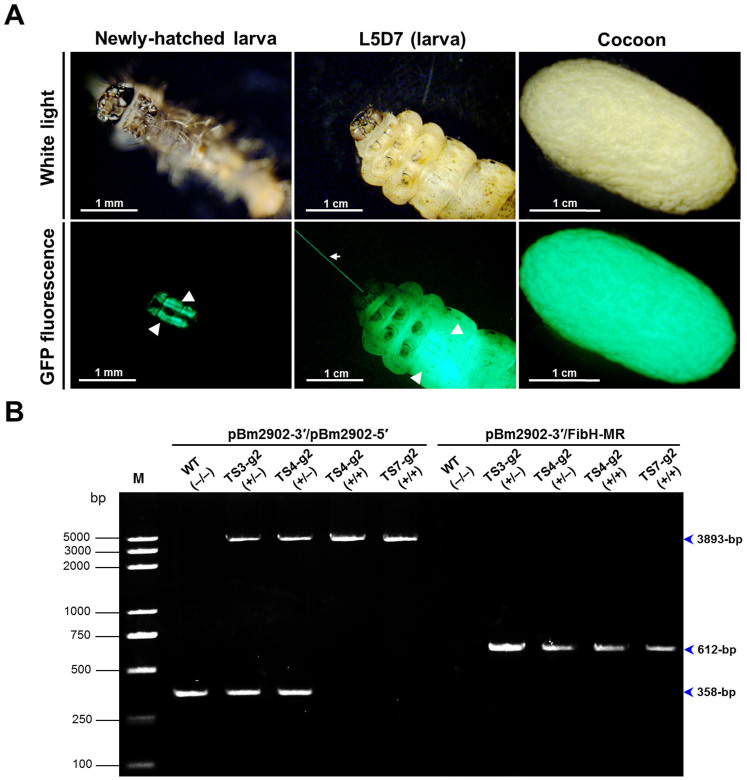
Genetic stability of transgene integration in TS3-g2 offspring. (A) Expression of the *EGFP* gene detected in newly-hatched larva, 7th day of the 5th instar larva (L5D7) and coccon from TS7-g2 silkworms. Triangles denote the position of the GFP fluorescence in the larval silk glands. Arrowhead denotes a single silk with GFP fluorescence from L5D7 larva. The different scale bars are located at the bottom left of the images. (B) PCR analysis of the genomic DNA using primer pairs pBm2902-3′/pBm2902-5′ and pBm2902-3′/FibH-MR. The left panel shows the PCR products of the genomic DNA from individuals of wild-type 871 (WT, −/−), TS3-g2 (+/−), TS4-g2 (+/−), TS4-g2 (+/+) and TS7-g2 (+/+) using primer pair pBm2902-3′/pBm2902-5′, and the right panel shows the PCR products of the genomic DNA from individuals of wild-type 871 (WT, −/−), TS3-g2 (+/−), TS4-g2 (+/−), TS4-g2 (+/+) and TS7-g2 (+/+) using primer pair pBm2902-3′/FibH-MR. Lane M, Trans2K Plus DNA Marker. “−/−”, “+/−” and “+/+” indicate the non-transgenic, heterozygous and homozygous transgenic DNA samples, respectively. Sizes of the PCR products are indicated on the right of each panel.

In addition, EGFP expression was detected in TS7-g2 individuals and their cocoons. As shown in Figure 6A, EGFP was highly efficiently and specifically expressed in the silk glands of the TS7-g2 silkworms and was spun into their cocoons. To further confirm the stability of transgene integration in the TS3-g2 offspring, PCR was performed on the genomic DNA from TS3-g2 (+/−), TS4-g2 (+/−), TS4-g2 (+/+), and TS7-g2 (+/+) individuals using primer pairs pBm2902-3′/pBm2902-5′ and pBm2902-3′/FibH-MR. As shown in Figure 6B, the PCR products were a 3893-bp DNA fragment for TS3-g2 (+/−), TS4-g2 (+/−), TS4-g2 (+/+), and TS7-g2 (+/+) individuals using the primer pair pBm2902-3′/pBm2902-5′, a 358-bp DNA fragment for wild-type 871 (WT, −/−, indicates a nontransgenic), TS3-g2 (+/−), and TS4-g2 (+/−) individuals using the primer pair pBm2902-3′/pBm2902-5′, and a 612-bp DNA fragment for TS3-g2 (+/−), TS4-g2 (+/−), TS4-g2 (+/+), and TS7-g2 (+/+) individuals using the primer pair pBm2902-3′/FibH-MR, which is consistent with the expected pattern. All the above results confirmed that the integration of FibH-EGFP expression cassette and the expression of EGFP were stable in the TS3 offspring whether PBase is present or absence *in vivo*.

## Discussion

To allow fine functional research into unknown genes and the establishment of a stable and efficient *B. mori* silk gland bioreactor, the disadvantages of the position effects and insertional mutagenesis caused by *piggyBac*-mediated random integration must be overcome. Therefore, we used a combination of different genomic manipulation techniques and the optimized fibroin H-chain expression system to develop a generic and efficient strategy for establishing a stable, replaceable, and highly efficient transgene expression system in the silkworm. To develop this strategy, we first inserted into the genomes of silkworms an exogenous target gene expression cassette that efficiently and selectively expresses the target protein in the silk glands of the silkworm, using the composite *piggyBac*-derived vector PB-TP, which was randomly integrated during the initial germline transformation. The structure of the PB-TP vector has been described above. It is noteworthy that this composite vector, with two full-length wild-type *piggyBac* arms R1 (1050 bp) and L1 (678 bp) and two shortened *piggyBac* arms L2 (309 bp) and R2 (238 bp), encodes four potential transposons (Supplementary Figure S1). It has been reported that the short *piggyBac* arm constructs can be used to improve the mobilization efficiency of *piggyBac* in the genomes of *D. melanogaster*^49^ and *B. mori*^50^. Therefore, L2 and R2 were developed to improve the efficiency of R1–L2 and R2–L1 remobilization in *B. mori in vivo*, thereby improving the deletion efficiency of R1–L2 and R2–L1 from the initial chromosomal insertional locus. Several previous studies have also demonstrated that not only the TIRs but also the flanking sequences of *piggyBac*, especially the TTAA sites, are required for its successful transposition^51^. Therefore, the TTAA sites were constructed at the 3′ end of the L2 and R2 sequences in the composite vector (shown in the primer sequences in Supplementary Table S1). To achieve the precise integration of other exogenous genes at the same genomic locus as the target gene with phiC31-mediated RMCE, two *attP* sites were introduced, flanking the target gene expression cassette, in the same orientation.

In this study, we established four different transgenic strains, TS1-RgG1–TS1-RgG4, with the random insertion of transposon R1–L1 into the silkworm genome, and the cocoons of the different TS1-RgG strains displayed pure EGFP contents ranging from 0.9% to 16% (w/w). This result indicates that a transgene inserted at different chromosomal loci greatly affects the expression of the exogenous protein in the silkworm. To our knowledge, TS1-RgG2 is so far the most efficient transgenic silkworm strain producing exogenous protein in the silk gland^18^,^20^,^52^,^53^,^54^,^55^. The subsequent elimination of all transposon sequences containing the PBase gene expression cassette and all the marker genes for TS2-RgG2 was completed with the heat-shock-induced expression of the transposase *in vivo*, generating TS3-g2, which contains only the *attP*-flanked optimized fibroin H-chain expression cassette in its genome. This method not only prevents the remobilization of the target gene, but also eliminates the adverse effects of the selectable marker genes in any future application of the transgenic insect, which may affect the expression of the target genes, the growth and development of the transgenic individuals, horizontal gene transfer, or any of the other potential ecological security problems associated with transgenic insects^21^. The sequencing results showed that there was no footprint or any structural changes, such as deletions, inversions, or rearrangements, at the excision-site TTAA elements or *attP* sites in the genomes of the TS3-g2 individuals. Further results confirmed the genetic stability of the integration and the expression of the *EGFP* gene in the TS3-g2 offspring. In a future study, different genes of interest will be placed precisely at the same genomic locus of the TS3-g2 silkworm using a phiC31-mediated RMCE reaction. This will achieve the highly efficient, stable expression of different exogenous proteins in the transgenic silkworms, mediated by the fibroin H-chain promoter. Because the phiC31-integrase-mediated recombination between the *attP* and *attB* sites is unidirectional, it ensures the stability of the transgenes after the RMCE reaction^56^. The TS3-g2 silkworms established in this study can be used to create a highly efficient transgenic silkworm silk gland bioreactor for the production of exogenous proteins. Importantly, they can also be used to improve the natural cocoon silks and produce novel silk fibers with high tensile strength, high adhesion, and other excellent properties with the silk-gland-specific expression of structurally related proteins (such as the spider dragline protein^57^,^58^). Actually, other silk-gland-specific promoters (such as the *FibL*^59^,^60^, *fhx*^52^, and *Ser1* promoters^61^) or tissue-specific promoters (such as fat-body-62, midgut-63 and hemocyte-specific promoters^64^) can also be used to establish stable, replaceable, and highly efficient transgene expression systems in the silkworm with this generic strategy (Figure 1). In addition, the FLP/*FRT* and Cre/*loxP* systems have been successful used for RMCE reactions in *D. melanogaster*^22^,^29^,^65^. Furthermore, Schetelig and Handler recently described a Cre-madiatied RMCE system that is highly efficient in *D. melanogaster*, and for the first time in a non-drosophilid, the tephritid fly, *Anastrepha suspensa*^66^. Compared with the phiC31-mediated RMCE system, FLP- and Cre-mediated RMCE have the main advantage that allowed for multiple insertion/deletion events of transgenes at a single locus^65^,^66^. In the future work, FLP- and Cre-mediated RMCE systems also could be combined with different genomic manipulation techniques described above, and introduced as a powerful tool for functional genomic comparisons and to develop the most advanced transgenic silkworm strains for applied use.

In this study, a mixture of the PB-TP vector and the helper plasmid pHA3PIG was injected into G0 eggs to create TS1 individuals with initial germline transformation. The transgenic individuals could also be generated by the injection of the PB-TP vector alone, without the helper plasmid, because the *Drosophila hsp70* promoter in the PB-TP vector induces highly efficient transient protein expression in the embryos of several different insect species, including *D. melanogaster*^10^, *Anopheles stephensi*^40^, and *B. mori*^67^. The structural combination of four different transposons is encoded in the composite PB-TP vector, but the germline transformation of only the R1–L1 construct was expected during the initial transformation. Therefore, a silkworm cytoplasmic A3-promoter-driven *Pbase* gene expression vector, pHA3PIG, was used as the helper plasmid for the production of PBase, which will increase the efficiency of the initial expected transformation, thereby enhancing the probability of producing TS1-RgG individuals.

Here, we also used the *hsp70* promoter to induce *PBase* gene expression in the TS2-RgG2 individuals *in vivo* with HST. The HST method is simpler than the direct injection method, and the main disadvantage of the sexual hybridization method is that the *PBase* gene sequence is introduced into the genome of the hybrid offspring, allowing the persistent expression of PBase, which can reduce the deletion efficiency of R1–L2 and R2–L1. Previous studies have shown that the *hsp70* promoter best induces the expression of downstream genes when silkworms are exposed to continuous and repeated HST at 42°C in their developmental stages^68^,^69^. The embryonic silkworm develops from a single-celled zygote to a larva, and the fourth instar larval stage of the silkworm is a critical period in the formation of its secondary spermatocytes or the development of its primary oocytes^70^. Therefore, continuous and repeated HST at 42°C was applied to treat the transgenic silkworms at the embryonic stage or fourth instar larval stage in this study. The results suggest that HST in the embryonic or larval stage affects the normal growth of the transgenic silkworms, and that HST in the larval stage causes the highest mortality rate (Table 4). Compared with the offspring of individuals in the non-HST control groups, the deletion efficiencies of R1–L2 and/or R2–L1 were significantly higher in the offspring of TS2-RgG2 individuals in the HST groups, especially in the offspring of groups with HST at the embryonic stage (Table 5). The deletion efficiency of R2–L1 was up to 80% in the offspring of TS4-gG-2 individuals with HST applied in the embryonic stage (Supplementary Table S3 and Figure 5A). All these results suggest that continuous and repeated HST at 42°C in the embryonic stage of transgenic silkworms is the most effective way to delete the transposons from their offspring.

However, a few G3 gG-positive broods from TS2-RgG2 individuals and G5 g-positive broods from TS4-gG individuals were also observed in the non-HST groups, which is attributable to the background expression of PBase under the control of the *hsp70* promoter, even though this background activity was very low in the transgenic silkworms *in vivo* (Table 5 and Supplementary Table S3). These results are consistent with those of a previous study that reported the background expression of a *Bombyx* nuclear receptor Ftz-F1 gene (*BmFtz-F1*) under the control of the *Drosophila hsp70* promoter in transgenic silkworms^68^. Although the basal activity of the *hsp70* promoter was low at 25°C, our study confirms that the *Drosophila hsp70* promoter is a very effective inducible promoter for regulating the expression of exogenous genes in transgenic silkworms.

**Figure 7:**
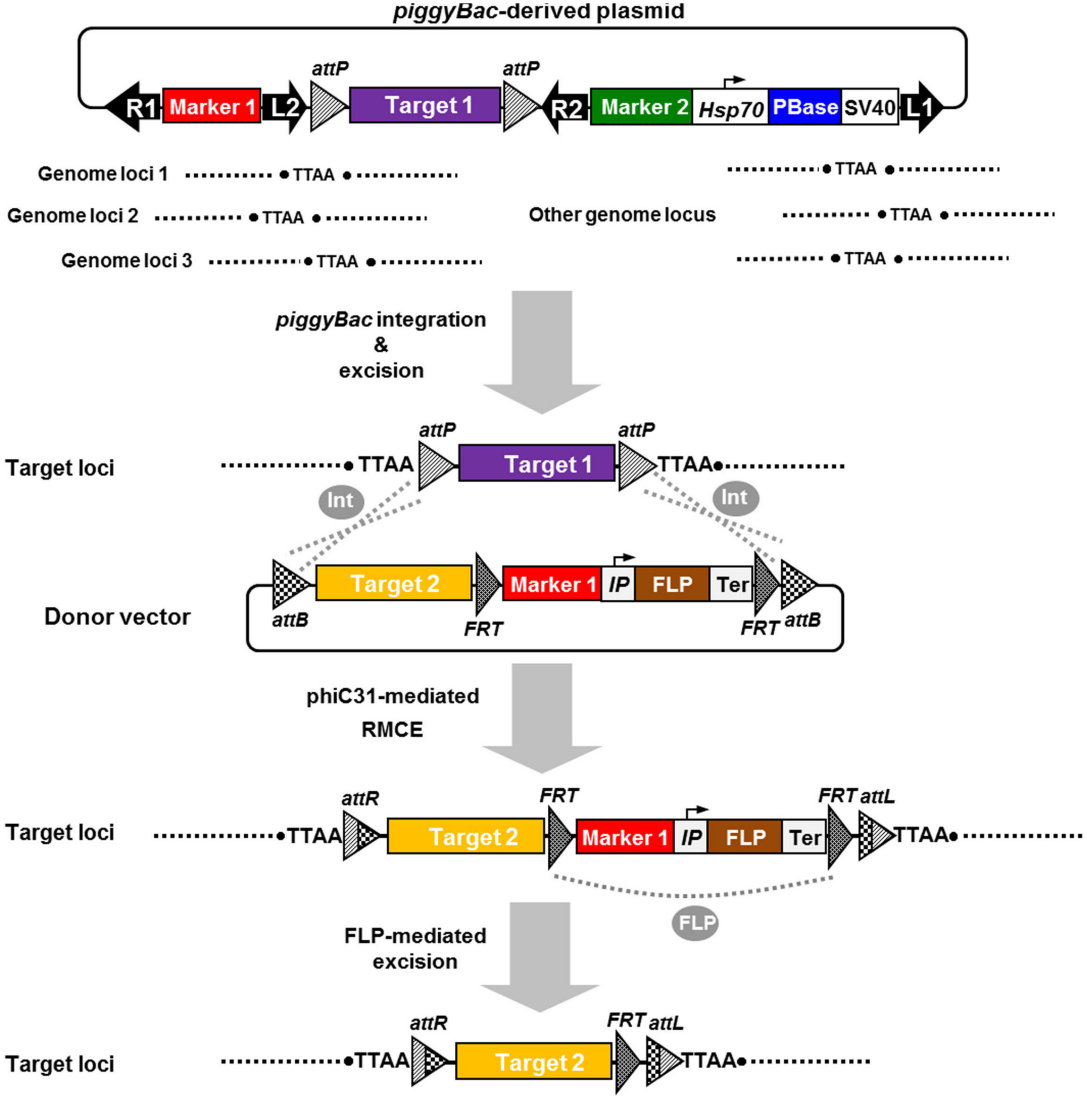
Strategy for site-specific gene integration via phiC31-mediated RMCE and subsequent marker excision from the target locus using the FLP/*FRT* system. Production and selection of transgenic silkworm strains contained a single copy of the target gene 1 (Traget 1) without any transposon and marker genes (Marker 1 and Marker 2) in their genomes (Target loci) using the *piggyBac*-derived plasmid and as described in Methods and shown in Figure 1. Recombination between two *attB*/*attP* pairs from donor vector and the target loci mediated by phiC31 integrase (Int) resulted in integration of a single copy of the taget gene 2 (Target 2), the Marker 1 and an inducible promoter (IP)-driving FLP recombinase expression cassette (Ter indicates the termination sequence) into the target loci of the silkworm genome. Subsequent inducible expression of FLP recombinase (FLP) *in vivo*, which can catalyze recombination between two *FRT* sites and resulting excision of the Marker 1 and the FLP recombinase expression cassette from the target loci of the silkworm genome.

In recent years, genome-editing methods, such as zinc finger nuclease (ZFN), transcription-activator-like effector nuclease (TALEN), and clustered regularly interspersed short palindromic repeats (CRISPR) RNA-guided Cas9 nuclease, have been successfully used to target and cleave genes in the silkworm^71^,^72^,^73^. However, the length of the DNA fragment integrated into the silkworm genome by TALEN-mediated gene editing using single-stranded DNA oligonucleotides is very limited^74^, and the only reported efficient *GFP* expression cassette (*A3-GFP-SV40T*) knock-in was still very limited in the silkworm when mediated by ZFN (just 0.008%, 1/11770)^75^. Figure 7 shows an efficient method for modifying previously inserted transgenes and for the integration of large DNA fragments into the silkworm genome using a combination of the *piggyBac*-based transposon-free method, the phiC31-mediated RMCE system, and the FLP/*FRT* system. The phiC31/*att* system has been shown allow the integration of DNA of up to 100 kb into specific recipient sites in *D. melanogaster*^76^, so this method could be used to overcome the disadvantages of these genome-editing systems. Furthermore, a combination of the SSR system and the genome-editing methods described above should overcome both the random insertion of transposons and the problems associated with the integration of large DNA fragments using genome-editing systems.

In conclusion, we have developed an efficient and generic strategy for producing a stable, replaceable, and highly efficient transgene expression system in *B. mori*. Our strategy effectively eliminates the remobilization of *piggyBac*-mediated integrated transgenes in the silkworm. It is also applicable to other lepidopteran insects to improve the ecological safety of transgenic strains intended for release in biocontrol programs. Because silkworms are a commercially important insect and are widely used as an experimental model of lepidopteran insects, the transgenic strains established in this study can be used not only to optimize exogenous protein expression and to improve the properties of natural cocoon silks, but also in functional genomic research, such as investigating the functions of different genes at the same locations in the silkworm genome. The use of the *piggyBac*-based transposon-free method combined with a phiC31-mediated RMCE system in *B. mori* or any other species has not been reported until now. In a future study, we will combine our strategy with the genome-editing systems described above to establish genomic manipulation technologies in the silkworm and other lepidopteran species.

## Methods

### Experimental animals

The Chinese lineage diapause *B. mori* strain 871 (white cocoon, commercial strain) is maintained at the Gene Resource Bank of Domesticated Silkworms, Southwest University, Chongqing, China. The 15°C-IMES germline transformation strategy developed by us^48^ was used to change the diapause character of the 871 eggs before DNA preblastoderm microinjection. After they were injected, the eggs were maintained at 25°C in a moist chamber (85%–90% relative humidity) until hatching. The larvae were reared at 25°C (75%–80% relative humidity) and fed mulberry leaves.

### Plasmid construction

The *piggyBac*-derived target plasmid vector pBac{R1-3×P3-DsRed-SV40-L2-*attP*-FibH-EGFP-LBS-*attP*-R2-3×P3-EGFP-SV40-Hsp70-PBase-SV40-L1} (designated “PB-TP”; Figure 1A) was constructed as described in the Supplementary Materials. The sequences of the primers used in the plasmid construction are shown in Supplementary Table S1. Briefly, (*i*) the *attP*-pBacR2 fragment was PCR amplified from pBac{3×P3-DsRedaf}^77^ and cloned into the plasmid pSL{3×P3-EGFP-SV40}^26^ to generate pSL{*attP*-R2-3×P3-EGFP-SV40}; (*ii*) the *Drosophila* *hsp70* promoter, the *PBase* gene, and the SV40 polyA were PCR amplified from the plasmids pMLS104^78^, pHA3PIG^13^, and pBac{3×P3-DsRedaf}, respectively, and the three amplified fragments were inserted into the plasmid pSLfa1180fa^77^ to generate pSL{Hsp70-PBase-SV40}; (*iii*) the Hsp70-PBase-SV40 fragment was SphI-digested from pSL{Hsp70-PBase-SV40} and cloned into the plasmid pSL{*attP*-R2-3×P3-EGFP-SV40} to generate pSL{*attP*-R2-3×P3-EGFP-SV40-Hsp70-PBase-SV40}; (*iv*) the pBacL2-*attP* fragment was PCR amplified from pBac{3×P3-DsRedaf} and inserted into the plasmid pBac{3×P3-DsRedaf}-R3^20^ to generate pBac{R1-3×P3-DsRed-SV40-L2-*attP*-FibH-EGFP-LBS-L1}; (*v*) the *attP*-R2-3×P3-EGFP-SV40-Hsp70-PBase-SV40 fragment was FseI-digested from pSL{*attP*-R2-3×P3-EGFP-SV40-Hsp70-PBase-SV40} and cloned into the plasmid pBac{R1-3×P3-DsRed-SV40-L2-*attP*-FibH-EGFP-LBS-L1} to generate the PB-TP vector.

### Germline transformation and marker detection

The germline of *B. mori* was transformed with a *piggyBac* vector, as previously described^26^,^28^. G0 nondiapause eggs from strain 871 were collected for microinjection within 2 h of oviposition. A 1:1 (volume ratio) mixture of 450 ng/μL PB-TP vector and 400 ng/μL helper plasmid pHA3PIG in superpure water was injected into each egg with a FemtoJet 5247 microinjector system (Eppendorf, Hamburg, Germany). The G0 embryos were allowed to develop at 25°C. The fertile G0 adults were backcrossed with wild-type 871 adults to produce G1 offspring.

The expression of 3×P3-DsRed and 3×P3-EGFP in the eyes and nervous systems of the G1 embryos, larvae, pupae, and adults was detected with an Olympus MacroViewMVX10-AUTO fluorescence stereomicroscope (Olympus, Tokyo, Japan) with a red fluorescent protein (RFP) or green fluorescent protein (GFP) filter, respectively. The expression of the EGFP/H-chain fusion protein (FibH-EGFP) in the silk gland of the G1 larvae and in the cocoon silk from G1 individuals was detected with the same fluorescence stereomicroscope and a GFP filter. Filters passing light at 510–550 nm for DsRed and at 460–490 nm for EGFP were used for excitation. Positive G1 larvae from different broods were reared (with each brood considered a unit), and the transgenic strains (TSs) were then produced.

### SDS-PAGE and immunoblotting analysis

SDS-PAGE and immunoblotting analysis of EGFP in the silkworm cocoon were performed as described in our previous report^79^. Briefly, about 25 mg of each silkworm cocoon was dissolved in 1 mL of 60% (w/v) lithium thiocyanate (LiSCN), and the 1 μg/μL GFP standard (Abcam, Cambridge, UK) was diluted fivefold with the same 60% LiSCN. The samples (1.5 μL) and the GFP standard were subjected to SDS-PAGE (12% [w/v] polyacrylamide slab gel) by dissolving them in equal volumes of sample loading buffer with 2% (v/v) β-mercaptoethanol (2-ME), and boiling them for 5 min. After electrophoresis, the gel was stained with Coomassie Brilliant Blue R-250. An aliquot (0.5 μL) of each sample and the GFP standard were dissolved in the sample loading buffer with 2% 2-ME and subjected to SDS-PAGE. The proteins were transferred directly from the gel onto a polyvinylidene difluoride membrane (Roche, Mannheim, Germany), which was then incubated at room temperature for 1 h with TBST containing 2500-fold diluted anti-GFP antibody (Beyotime, Jiangsu, China). The membrane was then incubated at room temperature for 1 h in TBST containing horseradish-peroxidase-labeled anti-rabbit IgG secondary antibody diluted 10,000-fold (Beyotime). The immunoreactive bands were visualized with the ECL Plus Western Blotting Detection Reagents, according to the manufacturer's instructions (Beyotime), and a chemiluminescence imaging system (Clinx ChemiScope series, Shanghai, China). The relative intensities of the bands were calculated with the ImageJ software and compared with that of the GFP standard used as the control.

### Southern blotting analysis

Genomic DNA was prepared from the silkworms using the procedure described by Zhao et al.^80^ A Southern blotting analysis was performed as described by Long et al.^28^ About 25 μg of genomic DNA was digested with the indicated restriction enzymes and blotted onto a Hybond-N^+^ nylon filter (Amersham Bioscience, Piscataway, NJ, USA) after agarose gel electrophoresis. A 678-bp *DsRed* gene fragment was amplified from pBac{3×P3-DsRedaf} with primers pDsRed-f and pDsRed-r, and a 720-bp *EGFP* gene fragment was amplified from pBac{3×P3-EGFPaf}^77^ with primers pEGFP-f and pEGFP-r (Supplementary Table S1). These two PCR products were labeled with the DIG High Prime DNA Labeling and Detection Starter Kit II (Roche, Mannheim, Germany) and used as probes.

### Inverse PCR analysis

The chromosomal insertion sites of the transgene constructs were determined with inverse PCR, as previously described^26^,^28^. About 10 μg of genomic DNA was digested with HaeIII overnight at 37°C and circularized by ligation overnight at 16°C. The ligated product was PCR amplified with the transposon-specific primer pairs PLF/PLR (for *piggyBac* left arm 1, L1) and PRF/PRR (for *piggyBac* right arm 1, R1) (Supplementary Table S1). The sequencing results were analyzed with the Silkworm Genome Database (SilkDB; http://www.silkdb.org/silkdb/). Localization of the silkworm genomic insertion sites of the transgene constructs was completed using the SilkMap software (www.silkdb.org/silksoft/silkmap.html).

### *piggyBac* remobilization

The flanking transposons in the TSs individuals were remobilized with heat shock treatments (HSTs), as described in detail in the Supplementary Materials. Briefly, (*i*) TS1-RgG adults were backcrossed with wild-type 871 adults to produce G2 eggs, and these eggs were treated with HCl solution to break the diapause; (*ii*) three-day-old G2 nondiapause eggs were heat shocked at 42°C for 60 min three times a day at 6 h intervals for five days, or day 0 fourth instar G2 larvae were heat shocked at 42°C for 60 min three times a day at 6 h intervals for three days; (*iii*) after heat shock, the G2 eggs or larvae were maintained at 25°C. TS2-RgG adults from these G2 individuals were selected and backcrossed to wild-type 871 adults. TS3-g individuals from newly hatched G3 larvae were screened (for both L1–R2 and L2–R1 deletion) under a fluorescence stereomicroscope, as described above, and these TS3-g individuals were reared to adulthood and sib-mated or backcrossed with wild-type 871 adults to generate offspring.

### PCR analysis

Extracted genomic DNA from the TSs and wild-type 871 silkworms was used as the templates for PCR. The primer sequences used in the PCR analysis are shown in Supplementary Table S1. The purified PCR fragments were cloned into the plasmid pMD19-T Simple (Takara, Dalian, China) and sequenced.

## Supplementary Material

Supplementary Information Supplementary information

## Figures and Tables

**Table 1:** Injection of PB-TP vector in G0 silkworm embryos of the strain 871

Experiment No.	Injected eggs	Hatched eggs (%)	G1 broods	Positive G1 broods	Percent of positive G1 broods, %
1	268	93 (34.70)	22	3	13.6
2	358	186 (51.96)	78	8	10.3
Total	626	279 (44.57)	100	11	11.0

**Table 2:** Analysis of different fluorescence phenotypes of TS1 individuals from positive G1 broods

Experiment No.	Fluorescence phenotype of TS1 individuals (broods)							Total number of TS1 individuals in one experiment (broods)
	TS1-RgG	TS1-Rg	TS1-gG	TS1-RG	TS1-R	TS1-g	TS1-G	
1	1 (1)	0	0	0	5 (1)	0	9 (3)	15 (3)
2	3 (3)	0	0	4 (1)	23 (6)	0	2 (1)	32 (8)
Total	4 (4)	0	0	4 (1)	28 (7)	0	11 (4)	47 (11)

TS1 individuals were identified from positive G1 broods by fluorescence phenotypes, that is, presence of which of the three markers they expressed: 3×P3-DsRed (R), FibH-EGFP (g) and/or 3×P3-EGFP (G). Numbers of TS1 individuals containing different fluorescence phenotypes are shown, with the number of broods containing at least one transgenic larva (designated the “positive brood”).

**Table 3:** Identification of genomic insertion sites of R1–L1 transgene construct in TS1-RgG individuals by inverse PCR

Strain	Scaffold	Chromosome^a^	5′-Genomic sequence^b^	3′-Genomic sequence^c^
TS1-RgG1	nscansf2891	24	AGCTCAC TGTCCACGTGGTG**TTAA**	**TTAA**GTGCTTATGGGAGCCCATAG
TS1-RgG2	nscaf2902	18	AGTCAGTCAGTCAAACATAT**TTAA**	**TTAA**GTATATTTGTTAATTTATAT
TS1-RgG3	scaffold16066^d^	–	TGAAGAGTGACGTCAAAGTT**TTAA**	**TTAA**TTGCATATTGCGTAGATTCT
TS1-RgG4			Not identified	

^a^Localization of silkworm genomic insertion sites of *piggyBac* vectors were completed using the SilkMap software (www.silkdb.org/silksoft/silkmap.html).

^b^Flanking genomic sequences obtained with insertion site TTAA on the *piggyBac* left arm.

^c^Flanking genomic sequences obtained with insertion site TTAA on the *piggyBac* right arm.

^d^scaffold16066 is an unplaced scaffold (It is not known which chromosome the scaffold16066 belongs to).

**Table 4:** Analysis of different fluorescence phenotypes of TS2 individuals obtained by crossing a heterozygous TS1-RgG2 male with a wild-type 871 female

Group	G2 eggs	Hatched eggs (%)^a^	Total G2 fertile moths (%)^b^	Total TS2 fertile moths	Fluorescence phenotype of TS2 individuals					
					TS2-RgG2	TS2-Rg2	TS2-gG2	TS2-R2	TS2-g2	TS2-G2
1#	140	138 (98.57)	121 (87.68)	59	54	0	3	2	0	0
2#	137	108 (78.83)	90 (83.33)	47	41	0	4	2	0	0
3#	138	133 (96.38)	98 (73.68)	54	51	0	2	1	0	0

^a^Percentage of (Number of hatched eggs)/(Number of G2 eggs).

^b^Percentage of (Number of total G2 fertile moths)/(Number of hatched eggs).

**Table 5:** Post-integration removal of the flanking *piggyBac* transposons in TS3 individuals obtained by crossing TS2-RgG2 moths with wild-type 871 moths

Group	TS2-RgG2 fertile moths	G3 broods	G3 broods containing TS3 larvae with different fluorescence phenotype (%)			Total (%)^a^
			Rg-positive broods	gG-positive broods	g-positive broods	
1^#^	54	48	0	2 (4.17)	0	2 (4.17)
2^#^	41	37	7 (18.92)	18 (48.65)	6 (16.22)	23 (62.16)
3^#^	51	45	3 (6.67)	6 (13.33)	1 (2.22)	7 (15.56)

Group 1^#^, 2^#^, and 3^#^ represent the individuals of G2 broods without HST, HST in the embryonic stage and HST in the larval stage, respectively (as described in Supplementary Figure S4B and Supplementary Methods). Rg-, gG- and g-positive broods represent the G3 broods containing at least one TS3-Rg2, TS3-gG2 and TS3-g2 larva, respectively.

^a^Total number of G3 broods containing at least one TS3-Rg2, TS3-gG2 or TS3-g2 larva.
