# Supplementary material for: An efficient strategy for producing a stable, replaceable, highly efficient transgene expression system in silkworm, Bombyx mori
Source: Sci Rep. 2015 Mar 5;5:8802. doi: 10.1038/srep08802 (PMC4350095; doi:10.1038/srep08802)
Supplement: Supplementary Information [file srep08802-s1.docx]

**Supplementary Data**

**An efficient strategy for producing a stable, replaceable, highly efficient transgene expression system in silkworm, *Bombyx mori***

Dingpei Long^1^, Weijian Lu^1^, Yuli Zhang^2^, Lihui Bi^2^, Zhonghuai Xiang^1^ and Aichun Zhao^1,*^

^1^State Key Laboratory of Silkworm Genome Biology, Key Laboratory for Sericulture Functional Genomics and Biotechnology of Agricultural Ministry, Southwest University, Chongqing 400716, People’s Republic of China and ^2^Guangxi Research Academy of Seficultural Science, Guangxi Nanning 530007, People’s Republic of China

* To whom correspondence should be addressed. Tel: +86-23-68251803; Fax: +86-23-68251128; E-mail: [zhaoaichun@hotmail.com](mailto:zhaoaichun@hotmail.com) or [zhaoaichun@swu.edu.cn](mailto:zhaoaichun@swu.edu.cn).

**Contents:**

**Supplementary Tables S1–S4**

**Supplementary Figures S1–S6**

**Supplementary Methods**

**Supplementary References**

**Supplementary Table S1.** Summary of primers used in present study

| Primer | Sequence (5′–3′)^a^ | Design basis |
| --- | --- | --- |
| attP-L2-F-AscI | tataggcgcgcc*CCCCCAACTGAGAGAACTCAAAGGTTACCCCAGTTGGGG*TTAACCCTAGAAAGATAGTCTGCG | *attP* site and *piggyBac* left arm sequence |
| L2-R-AscI | tataggcgcgccACGATATCTATAACAAGAAAAT | *piggyBac* left arm sequence |
| attP-R2-F-SpeI | ttaactagt*CCCCAACTGGGGTAACCTTTGAGTTCTCTCAGTTGGGGG*TTAACCCTAGAAAGATAATCATAT | *attP* site *and piggyBac* right arm sequence |
| R2-R-XhoI | ggacctcgagTAAAAGTTTTGTTACTTTATAG | *piggyBac* right arm sequence |
| SV40-F-NotI | gactgcggccgcGACTCTAGATCATAATCAGCC | SV40 polyadenylation signal sequence |
| SV40-R-SphI | gactgcatgcTACGCGTATCGATAAGCTTTAAG | SV40 polyadenylation signal sequence |
| Hsp70-F-SphI/SpeI | tataactagtgcatgcCTAGAATCCCAAAACAAACT | *D. melanogaster hsp70* promoter sequence |
| Hsp70-R-SpeI | ggacactagtTATTCAGAGTTCTCTTCTTGTAT | *D. melanogaster hsp70* promoter sequence |
| PBase-F-SpeI | tataactagtATGGGTAGTTCTTTAGACG | *piggyBac* transposase gene sequence |
| PBase-R-NotI | tatagcggccgCTAGAAACAACTTTGGCACATATC | *piggyBac* transposase gene sequence |
| pAmp-F | ATGAGTATTCAACATTTCCGTGTCG | Ampicillin resistance gene sequence |
| pAmp-R | TTACCAATGCTTAATCAGTGAGGC | Ampicillin resistance gene sequence |
| PLF | CTTGACCTTGCCACAGAGGACTATTAGAGG | *piggyBac* left arm sequence |
| PLR | CAGTGACACTTACCGCATTGACAAGCACGC | *piggyBac* left arm sequence |
| PRF | CGATAAAACACATGCGTCAATTTTACGC | *piggyBac* right arm sequence |
| PRR | GCTCTCGACAAATAACTTTTTTGCAT | *piggyBac* right arm sequence |
| pUC-F | TTAAAGTTTAGGTCGAGTAAAGCGC | *piggyBac* vector backbone sequence |
| FibH-MR | TGCAGGAGGTGATAGCTTGGGTC | *B. mori* fibroin H-chain gene promoter sequence |
| LBS-MF | GCTTCATCTCGCAGTTACGACTATTC | L-chain binding site of the *B. mori* fibroin H-chain gene sequence |
| pBm2902-5′ | TACACACATTTATGTATATCACAAAAAGCG | *B. mori* genome sequence |
| pBm2902-3′ | TACCGATTGATTGCATCTACG | *B. mori* genome sequence |
| pEGFP-f | ATGGTGAGCAAGGGCGAGG | Enhanced green fluorescent protein (EGFP) gene coding sequence |
| pEGFP-r | CTACTTGTACAGCTCGTCCATGCCG | Enhanced green fluorescent protein (EGFP) gene coding sequence |
| pDsRed-f | ATGGTGCGCTCCTCCAAGAACGT | Red fluorescent protein (DsRed) gene coding sequence |
| pDsRed-r | CAGGAACAGGTGGTGGCG | Red fluorescent protein (DsRed) gene coding sequence |

^a^The underlined sequences indicate the restriction enzyme cutting sites; The sequence of *attP* site was highlighted by *italics*; Quadrangular box indicates the “TTAA” site behind the *piggyBac* arm sequences.

**Supplementary Table S2.** Identification of genomic insertion sites of the transgene constructs in TS2 individuals from the G2 brood obtained by crossing a TS1-RgG2 male with a wild-type 871 famale

| Strain | Scaffold | Chromosome^a^ | 5′-Genomic sequence^b^ | 3′-Genomic sequence^c^ |
| --- | --- | --- | --- | --- |
| TS2-RgG2 | nscaf2902 | 18 | AGTCAGTCAGTCAAACATAT**TTAA** | **TTAA**GTATATTTGTTAATTTATAT |
| TS2-gG2 | nscaf2902 | 18 | AGTCAGTCAGTCAAACATAT**TTAA** | Not identified |
| TS2-R2 | nscaf2853 | 6 | Not identified | **TTAA**AGTATGCTGTATTAAAGTTC |

^a^Localization of silkworm genomic insertion sites of *piggyBac* vectors were completed using the SilkMap software ([www.silkdb.org/silksoft/silkmap.html](http://www.silkdb.org/silksoft/silkmap.html)).

^b^Flanking genomic sequences obtained with insertion site TTAA on the *piggyBac* left arm.

^c^Flanking genomic sequences obtained with insertion site TTAA on the *piggyBac* right arm.

**Supplementary Table S3.** Analysis of post-integration removal of the R2–L1 in TS3-gG2 offspring using different HST strategies

| Group | Crossing (♂×♀) | Screened G5 broods | G5 g-positive broods (%)^a^ | Frequencies of TS5-g2 individuals from each of the G5 g-positive broods, %^b^ | Frequencies of G5 individuals with g fluorescence phenotype from each brood, %^c^ |
| --- | --- | --- | --- | --- | --- |
| 1^#^ | TS3-gG2♂×871♀(a) | 50 | 1 (2) | 1.53 | 49.11–50.84 |
|  | TS3-gG2♂×871♀(b) | 50 | 1 (2) | 2.12 | 48.96–51.25 |
|  | TS3-gG2♂×871♀(c) | 50 | 0 | 0 | 49.79–50.76 |
| 2^#^ | TS3-gG2♂×871♀(a) | 50 | 37 (74) | 4.62–22.65 | 49.04–50.47 |
|  | TS3-gG2♂×871♀(b) | 50 | 35 (70) | 5.24–20.5 | 49.18–50.81 |
|  | TS3-gG2♂×871♀(c) | 50 | 40 (80) | 3.91–25.78 | 49.58–51.03 |
| 3^#^ | TS3-gG2♂×871♀(a) | 50 | 5 (10) | 3.16–5.38 | 49.16–50.29 |
|  | TS3-gG2♂×871♀(b) | 50 | 7 (14) | 3.31–5.81 | 48.85–51.03 |
|  | TS3-gG2♂×871♀(c) | 50 | 5 (10) | 2.61–5.56 | 49.52–50.82 |

Group 1^#^, 2^#^, and 3^#^ represent the individuals of G4 broods without HST, HST in the embryonic stage and HST in the larval stage, respectively (the same as described in Supplementary Figure S4B and Supplementary Methods). The larvae from 50 G5 broods of each group were analyzed for fluorescence phenotypes. Details of crossing strategies are illustrated and described in Supplementary Figure S6.

^a^Percentage of (Number of G5 g-positive broods)/(Number of screened G5 broods).

^b^Percentage of (Number of TS5-g2 individuals from one G5 g-positive brood)/(Total number of G5 individuals from this G5 g-positive brood). Each G5 g-positive brood contains 450–550 G5 individuals.

^c^G5 individuals with g fluorescence phenotype including TS5-gG2 and TS5-g2 individuals. Each G5 brood contains 450–550 G5 individuals.

**Supplementary Table S4.** The proportion of TS5-g2 individuals in each of the G5 broods obtained by reciprocal crosses between TS4-g2 adults and wild-type 871 adults

| Heterozygous TS4-g2 (+/–)^a^ adults × wild-type 871 (–/–)^b^ adults | | | | |  | Homozygous TS4-g2 (+/+)^c^ adults × wild-type 871 (–/–) adults | | | | |
| --- | --- | --- | --- | --- | --- | --- | --- | --- | --- | --- |
| No. | Crossing (♂×♀) | G5 eggs | Hatched eggs (%) | TS5-g2 individuals (%)^d^ |  | No. | Crossing (♂×♀) | G5 eggs | Hatched eggs (%) | TS5-g2 individuals (%) |
| 1 | TS4-g2♂×871♀ | 513 | 510 (99.42) | 253 (49.61) |  | 9 | TS4-g2♂×871♀ | 487 | 484 (99.38) | 484 (100) |
| 2 | TS4-g2♂×871♀ | 532 | 528 (99.25) | 260 (49.24) |  | 10 | TS4-g2♂×871♀ | 520 | 520 (100) | 520 (100) |
| 3 | TS4-g2♂×871♀ | 517 | 517 (100) | 262 (50.68) |  | 11 | TS4-g2♂×871♀ | 498 | 497 (100) | 497 (100) |
| 4 | TS4-g2♂×871♀ | 496 | 494 (99.6) | 243 (49.19) |  | 12 | TS4-g2♂×871♀ | 538 | 531 (98.7) | 531 (100) |
| 5 | 871♂×TS4-g2♀ | 484 | 484 (100) | 249 (51.45) |  | 13 | 871♂×TS4-g2♀ | 521 | 518 (99.42) | 518 (100) |
| 6 | 871♂×TS4-g2♀ | 524 | 523 (99.81) | 262 (50.1) |  | 14 | 871♂×TS4-g2♀ | 474 | 470 (99.16) | 470 (100) |
| 7 | 871♂×TS4-g2♀ | 509 | 509 (100) | 258 (50.69) |  | 15 | 871♂×TS4-g2♀ | 513 | 513 (100) | 513 (100) |
| 8 | 871♂×TS4-g2♀ | 518 | 516 (99.61) | 262 (50.78) |  | 16 | 871♂×TS4-g2♀ | 529 | 526 (99.43) | 526 (100) |
| Total |  | 4093 | 4081 (99.71) | 2049 (50.21) |  | Total |  | 4080 | 4059 (99.49) | 4059 (100) |

^a^+/–, indicates a heterozygote.

^b^–/–, indicates a nontransgenic.

^c^+/+, indicates a homozygote.

^d^Percentage of (Number of TS5-g individuals)/(Number of hatched eggs).

**A**

**
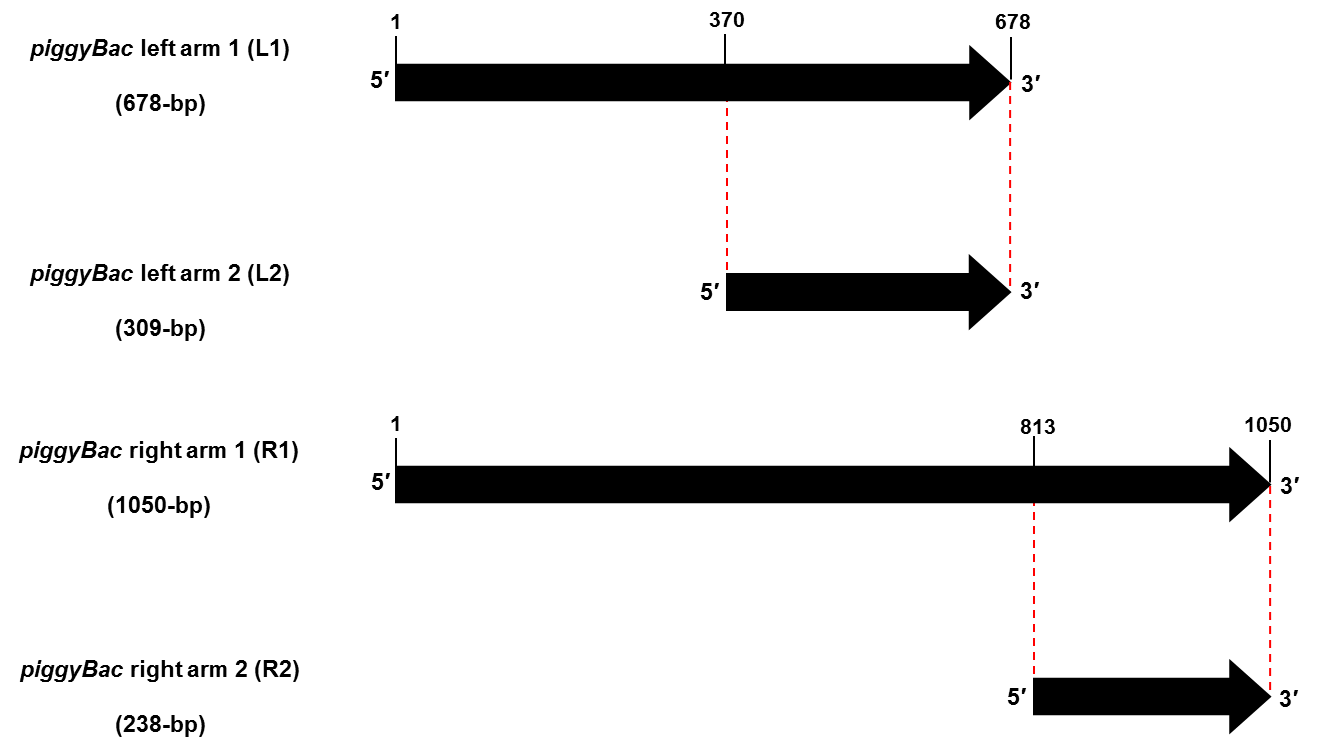
**

**B**

**>*piggyBac* left arm 1 (L1)**

GATCTGACAATGTTCAGTGCAGAGACTCGGCTACGCCTCGTGGACTTTGAAGTTGACCAACAATGTTTATTCTTACCTCTAATAGTCCTCTGTGGCAAGGTCAAGATTCTGTTAGAAGCCAATGAAGAACCTGGTTGTTCAATAACATTTTGTTCGTCTAATATTTCACTACCGCTTGACGTTGGCTGCACTTCATGTACCTCATCTATAAACGCTTCTTCTGTATCGCTCTGGACGTCATCTTCACTTACGTGATCTGATATTTCACTGTCAGAATCCTCACCAACAAGCTCGTCATCGCTTTGCAGAAGAGCAGAGAGGATATGCTCATCGTCTAAAGAACTACCCATTTTATTATATATTAGTCACGATATCTATAACAAGAAAATATATATATAATAAGTTATCACGTAAGTAGAACATGAAATAACAATATAATTATCGTATGAGTTAAATCTTAAAAGTCACGTAAAAGATAATCATGCGTCATTTTGACTCACGCGGTCGTTATAGTTCAAAATCAGTGACACTTACCGCATTGACAAGCACGCCTCACGGGAGCTCCAAGCGGCGACTGAGATGTCCTAAATGCACAGCGACGGATTCGCGCTATTTAGAAAGAGAGAGCAATATTTCAAGAATGCATGCGTCAATTTTACGCAGACTATCTTTCTAGGG

**>*piggyBac* left arm 2 (L2)**

GATATCTATAACAAGAAAATATATATATAATAAGTTATCACGTAAGTAGAACATGAAATAACAATATAATTATCGTATGAGTTAAATCTTAAAAGTCACGTAAAAGATAATCATGCGTCATTTTGACTCACGCGGTCGTTATAGTTCAAAATCAGTGACACTTACCGCATTGACAAGCACGCCTCACGGGAGCTCCAAGCGGCGACTGAGATGTCCTAAATGCACAGCGACGGATTCGCGCTATTTAGAAAGAGAGAGCAATATTTCAAGAATGCATGCGTCAATTTTACGCAGACTATCTTTCTAGGG

**>*piggyBac* right arm 1 (R1)**

AACCATTGTGGGAACCGTGCGATCAAACAAACGCGAGATACCGGAAGTACTGAAAAACAGTCGCTCCAGGCCAGTGGGAACATCGATGTTTTGTTTTGACGGACCCCTTACTCTCGTCTCATATAAACCGAAGCCAGCTAAGATGGTATACTTATTATCATCTTGTGATGAGGATGCTTCTATCAACGAAAGTACCGGTAAACCGCAAATGGTTATGTATTATAATCAAACTAAAGGCGGAGTGGACACGCTAGACCAAATGTGTTCTGTGATGACCTGCAGTAGGAAGACGAATAGGTGGCCTATGGCATTATTGTACGGAATGATAAACATTGCCTGCATAAATTCTTTTATTATATACAGCCATAATGTCAGTAGCAAGGGAGAAAAGGTTCAAAGTCGCAAAAAATTTATGAGAAACCTTTACATGAGCCTGACGTCATCGTTTATGCGTAAGCGTTTAGAAGCTCCTACTTTGAAGAGATATTTGCGCGATAATATCTCTAATATTTTGCCAAATGAAGTGCCTGGTACATCAGATGACAGTACTGAAGAGCCAGTAATGAAAAAACGTACTTACTGTACTTACTGCCCCTCTAAAATAAGGCGAAAGGCAAATGCATCGTGCAAAAAATGCAAAAAAGTTATTTGTCGAGAGCATAATATTGATATGTGCCAAAGTTGTTTCTGACTGACTAATAAGTATAATTTGTTTCTATTATGTATAAGTTAAGCTAATTACTTATTTTATAATACAACATGACTGTTTTTAAAGTACAAAATAAGTTTATTTTTGTAAAAGAGAGAATGTTTAAAAGTTTTGTTACTTTATAGAAGAAATTTTGAGTTTTTGTTTTTTTTTAATAAATAAATAAACATAAATAAATTGTTTGTTGAATTTATTATTAGTATGTAAGTGTAAATATAATAAAACTTAATATCTATTCAAATTAATAAATAAACCTCGATATACAGACCGATAAAACACATGCGTCAATTTTACGCATGATTATCTTTAACGTACGTCACAATATGATTATCTTTCTAGGG

**>*piggyBac* right arm 2 (R2)**

TAAAAGTTTTGTTACTTTATAGAAGAAATTTTGAGTTTTTGTTTTTTTTTAATAAATAAATAAACATAAATAAATTGTTTGTTGAATTTATTATTAGTATGTAAGTGTAAATATAATAAAACTTAATATCTATTCAAATTAATAAATAAACCTCGATATACAGACCGATAAAACACATGCGTCAATTTTACGCATGATTATCTTTAACGTACGTCACAATATGATTATCTTTCTAGGG

**Supplementary Figure S1.** The constructs (A) and complete sequences (B) of the *piggyBac* arms used in this study.

**
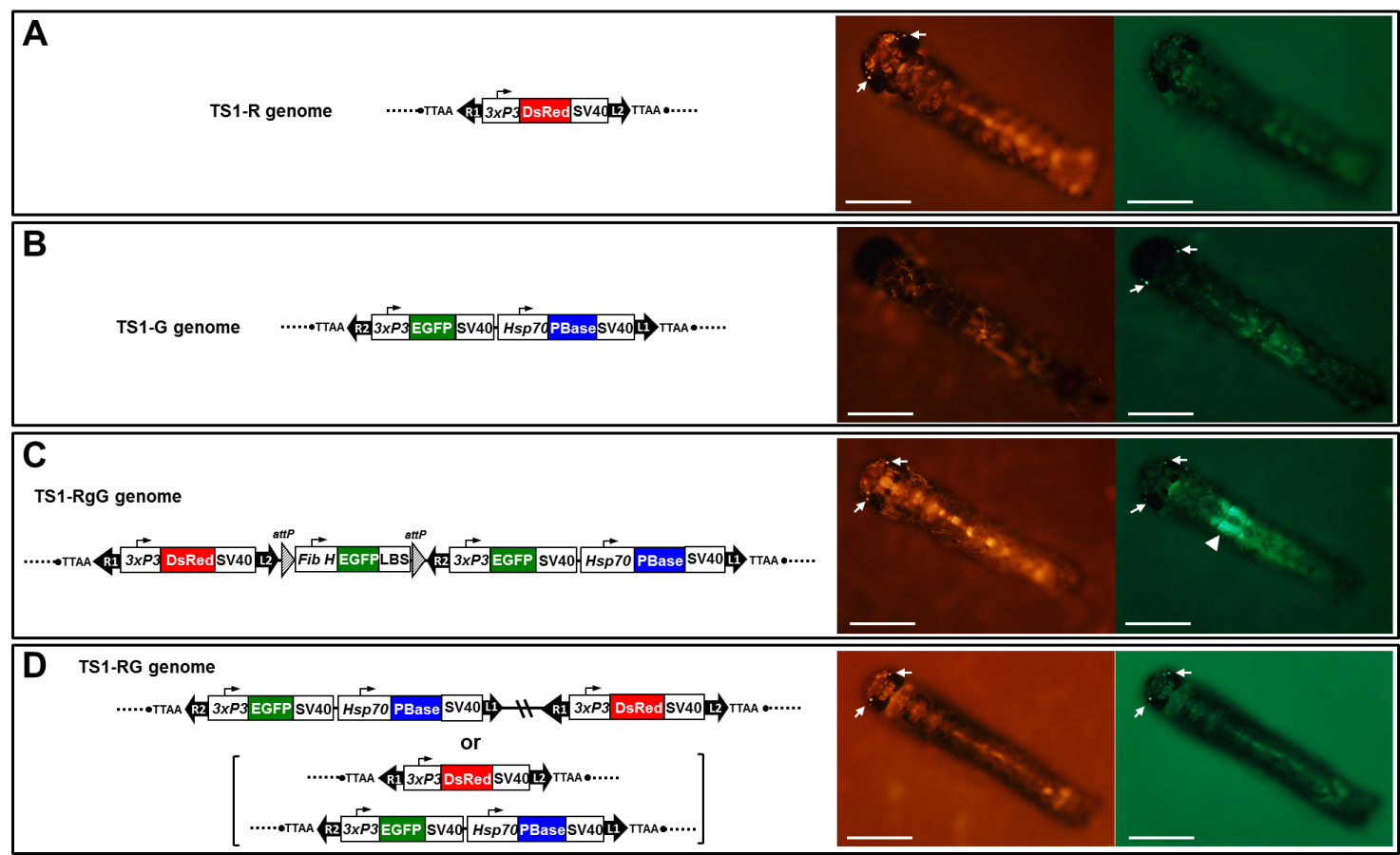
**

**Supplementary Figure S2.** Schematic maps of different transposons insertion in genomes of TS1 silkworms and expression of different fluorescent markers in TS1 silkworms. (A–D) show schematic of differnent types of transposons inserted into the TS1 genomes (*left*) and corresponding fluorescent phenotypes of newly-hatched TS1 larvae from R1–L2 (A), R2–L1 (B), R1–L1 (C), L2–R2 (or both of R1–L2 and R2–L1) (D) insertion (*right*). The expression of 3×P3-DsRed and/or 3×P3-EGFP markers in larval ocelli are highlighted with an arrowhead (A–D), and the expression of FibH-EGFP marker in larval silk glands are highlighted with a triangle (C). White scale bar represents 1 mm.

**
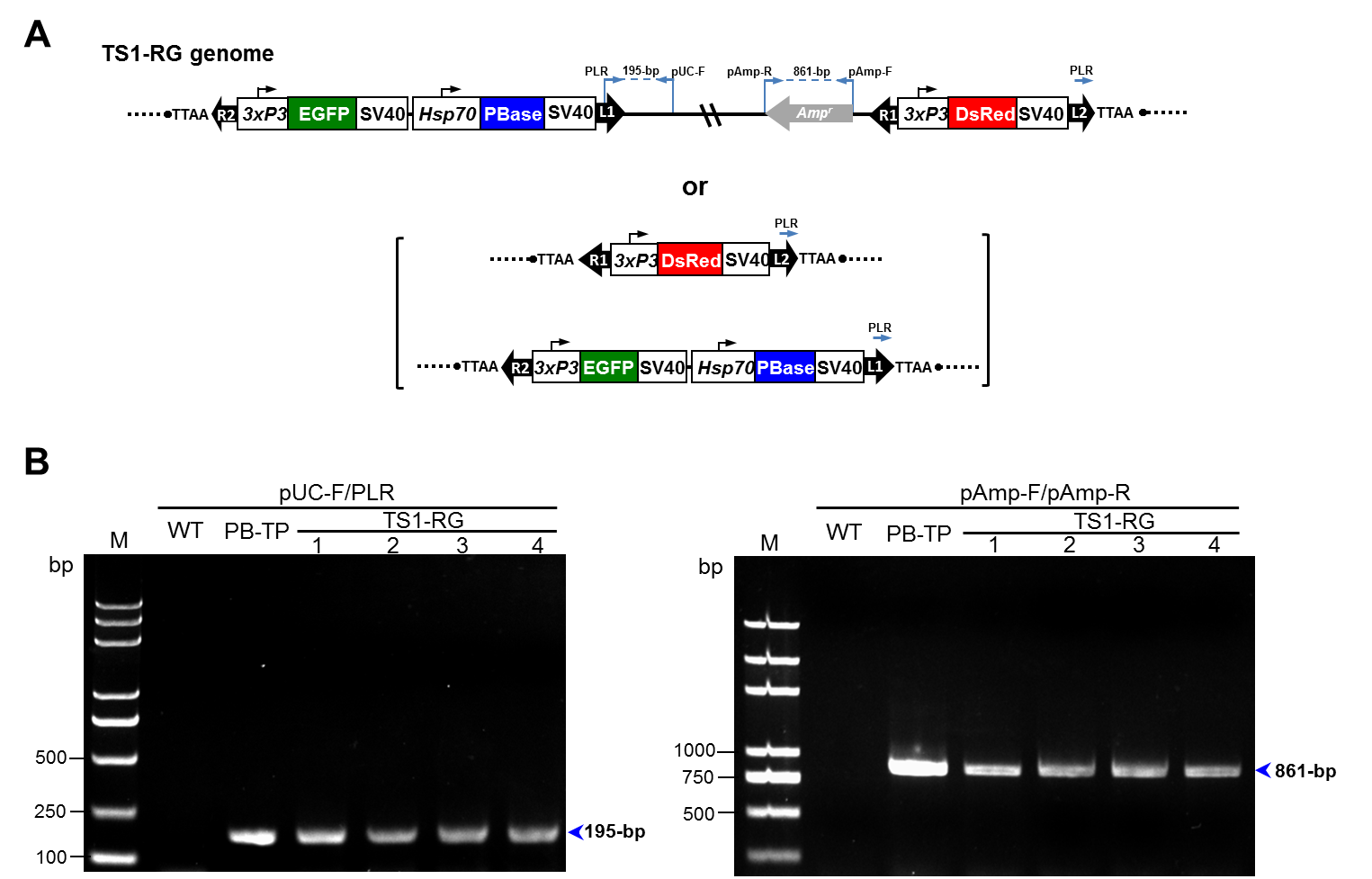
**

**Supplementary Figure S3.** Analysis of the insertion type of transposons in TS1-RG individuals. (**A**) Schematic maps of the L2–R2 (*top*) or both of R1–L2 and R2–L1 (*bottom*) constructs in genomes of TS1-RG individuals. Primers pUC-F, pAmp-F and pAmp-R were each designed from the backbone and ampicillin resistance gene (abbreviated *Amp^r^*) sequences of PB-TP vector (Supplementary Table S1). pUC-F/PLR and pAmp-F/pAmp-R primer pairs were used for PCR analysis of genomic DNAs from TS1-RG, respectively. (**B**) PCR analysis of genomic DNA using primer pairs pUC-F/PLR (*left*) and pAmp-F/pAmp-R (*right*) for the TS1-RG genomes. The PCR products were a 195-bp DNA fragment using primer pair pUC-F/PLR and an 861-bp DNA fragment using primer pair pAmp-F/pAmp-R for four TS1-RG individuals (lanes 1–4), which was consistent with the expected pattern of the L2–R2 insertion. Lane PB-TP, PB-TP vector used as a positive control; Lane WT, wild-type 871 strain used as a negative control; Lane M, Trans2K Plus DNA Marker.


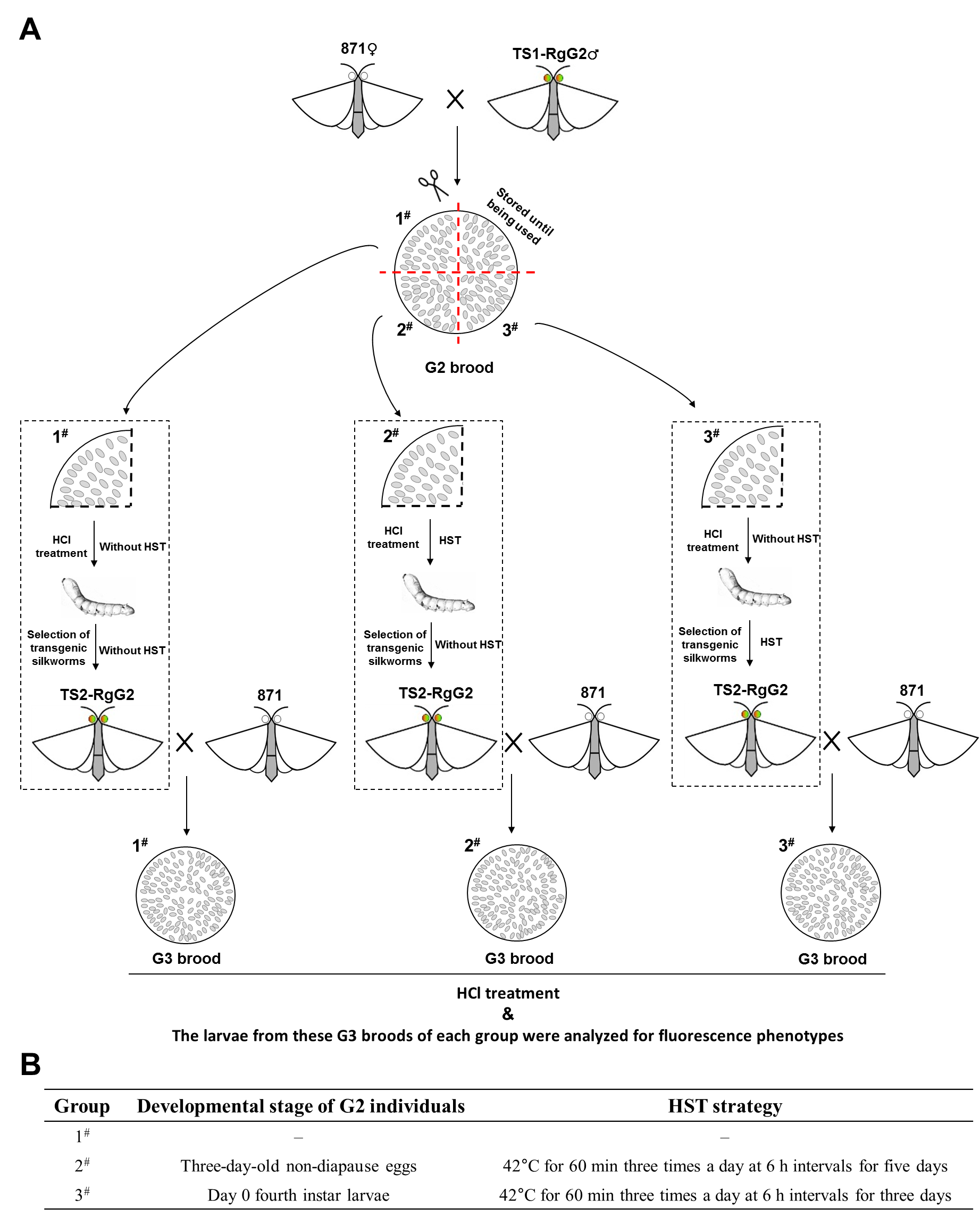


**Supplementary Figure S4.** Procedure for production of positive transposon-free silkworms. (A) Crossing strategies for production of G3 individuals for fluorescence detection. One TS1-RgG2 male (♂) heterozygous was backcrossed with wild-type 871 female (♀) to produce one G2 brood. This G2 brood was divided into four groups, and one group of the G2 diapause brood was stored until used. The G2 eggs from the other three groups (numbered 1^#^, 2^#^, and 3^#^) of this brood were treated with HCl solution to break the diapause. Then the individuals from the three groups of the G2 silkworms without or with heat shock treatment (HST), and the transgenic individuals were screened from these G2 individuals of each group. TS2-RgG2 fertile adults were backcrossed with adults from the wild-type 871 strain to produce G3 broods. G3 broods were treated with HCl solution to break the diapause, and the fluorescence phenotypes of the larvae from these G3 broods of each group were analyzed. (B) Different HST strategies for the individuals in different groups from the same G2 brood. Details of the HST strategies are described in the Supplementary Methods.


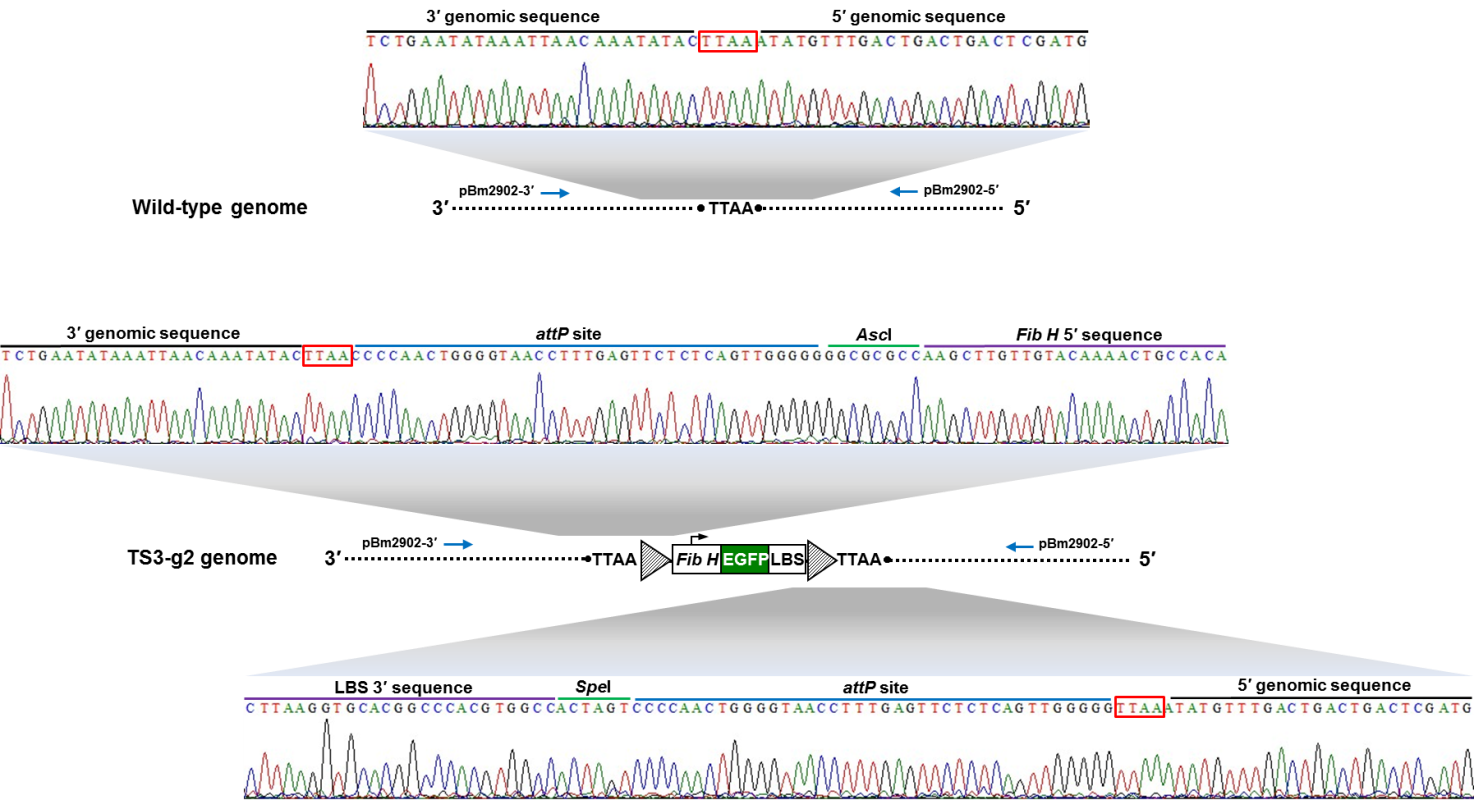


**Supplementary Figure S5.** Sequencing analysis of the genomic PCR products form wild-type 871 and TS3-g2 individuals. Compared with the sequence of the PCR product in wild-type genome (*top*), the *attP*-flanking FibH-EGFP-LBS expression cassette in the TS3-g2 genome (*bottom*) was as expected for a precisely *piggyBac* transposon excises without leaving a footprint at the excised site TTAA elements, and no structural changes were detected either in the cassette itself or in TS3-g2 genomic DNA.


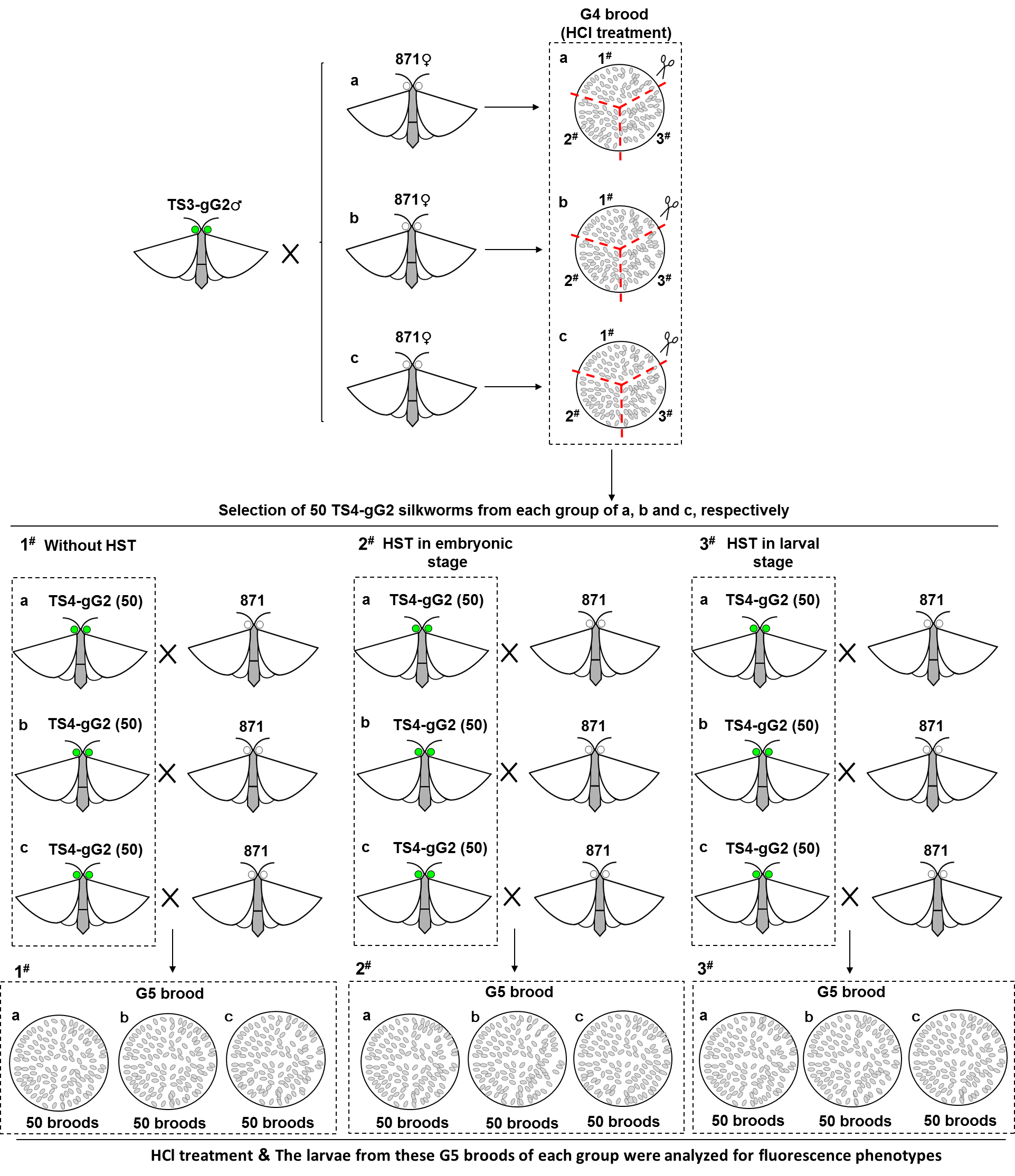


**Supplementary Figure S6.** Crossing strategies for production of G5 individuals for fluorescence detection. One TS3-gG2 male (♂) heterozygous was backcrossed with three different wild-type 871 females (♀) to produce three G4 broods (a, b, and c). The G4 eggs from each brood were divided into three groups (1^#^, 2^#^, and 3^#^) and treated with HCl solution to break the diapause. Then the individuals from the three groups of each G4 brood were treated with or without HST, as described in Supplementary Figure S4B and Supplementary Methods. 50 TS4-gG2 individuals were screened from a, b, and c broods of each group, respectively, and these TS4-gG2 fertile adults were backcrossed with adults from the wild-type 871 strain to produce G5 broods. These G5 broods were treated with HCl solution to break the diapause, and the fluorescence phenotypes of the larvae from 50 G5 broods of each group (G5 a, b and c broods) were analyzed.

**Supplementary Methods**

**Construction of target plasmid vector**

The PB-TP vector was constructed as described below. A 0.3-kb *attP*-pBacR2 fragment was amplified by PCR from pBac{3×P3-DsRedaf}^77^ with the primer pair *attP*-R2-F-SpeI and R2-R-XhoI. The amplified fragment was double-digested with SpeI and XhoI, and inserted into the SpeI/XhoI site of the plasmid pSL{3×P3-EGFP-SV40}^26^ to generate pSL{*attP*-R2-3×P3-EGFP-SV40}. The 0.27-kb SV40 polyadenylation signal sequence (SV40 polyA) was amplified by PCR using the primer pair SV40-F-NotI and SV40-R-SphI with the pBac{3×P3-DsRedaf} plasmid as a template. The PCR product was inserted into the NotI/SphI site of the plasmid pSLfa1180fa^77^ to generate pSL-SV40 polyA. The *piggyBac* transposase gene (*PBase*) was also PCR-amplified from the plasmid pHA3PIG^13^ with a PBase-F-SpeI and PBase-R-NotI primer pair, and the 1.8-kb PCR product was inserted into the SpeI/NotI site of the plasmid pSL-SV40 polyA to generate pSL-PBase-SV40. The 0.48-kb *Drosophila* heat shock protein 70 promoter (*hsp70*) was PCR-amplified from the plasmid pMLS104^78^ with an Hsp70-F-SphI/SpeI and Hsp70-R-SpeI primer pair. The amplified fragment was digested with SpeI, and inserted into the plasmid pSL-PBase-SV40 to generate pSL{Hsp70-PBase-SV40}. Then, the 2.32-kb Hsp70-PBase-SV40 fragment was recovered by digestion with SphI from pSL{Hsp70-PBase-SV40}, and inserted into the plasmid pSL{*attP*-R2-3×P3-EGFP-SV40} to generate pSL{*attP*-R2-3×P3-EGFP-SV40-Hsp70-PBase-SV40}. A 0.37-kb pBacL2-*attP* fragment was amplified by PCR from pBac{3×P3-DsRedaf} with the primer pair *attP*-L2-F-AscI and L2-R-AscI. The amplified fragment was digested with AscI, and inserted into the plasmid pBac{3xP3-DsRedaf}-R3^20^ to generate pBac{R1-3×P3-DsRed-SV40-L2-*attP*-FibH-EGFP-LBS-L1}. Finally, the PB-TP vector was generated by cloning a 4.23-kb *attP*-R2-3×P3-EGFP-SV40-Hsp70-PBase-SV40 FseI-digested fragment from the plasmid pSL{*attP*-R2-3×P3-EGFP-SV40-Hsp70-PBase-SV40} into the plasmid pBac{R1-3×P3-DsRed-SV40-L2-*attP*-FibH-EGFP-LBS-L1}.

The sequences of the PCR products and resulting recombinant plasmids were confirmed by sequencing.

**Heat shock treatment strategies for *piggyBac* remobilization**

Remobilization of the flanking transposons in the TSs individuals was done by heat shock treatments (HSTs) as described below. Firstly, TS1-RgG males were selected and backcrossed with wild-type 871 females to produce G2 eggs. For breaking diapause, 20-hour-old G2 eggs were treated with HCl solution (specific gravity, 1.075) at 46°C for 5 min, washed thoroughly with water and kept at 25°C. Then, two HST strategies for G2 non-diapause eggs or G2 larvae as follow:

Strategy 1, three-day-old G2 non-diapause eggs were placed in a plastic petri dish; the dish was placed in artificial climate incubator and the eggs were heat shocked for 60 min at 42°C (85%–90% relative humidity); following heat shock eggs were returned to 25°C (85%–90% relative humidity) for 6 h, then heat shocked again as the same way, three times a day for five days; after heat shock, the eggs were maintained at 25°C until hatching, and the hatched larvae were no longer in HST.

Strategy 2, G2 non-diapause eggs without HST; day 0 fourth instar G2 larvae were placed in a cardboard box; the box was placed in artificial climate incubator and the larvae were heat shocked for 60 min at 42°C (75%–80% relative humidity); following heat shock larvae were returned to 25°C (75%–80% relative humidity) and fed with mulberry leaves for 6 h, then heat shocked again as the same way, three times a day for three days; after heat shock, the larvae were reared at 25°C and fed with mulberry leaves.

Some of the G2 individuals without HST during their whole growth stage were used as control.

**Supplementary References**

13. Tamura, T. *et al.* Germline transformation of the silkworm *Bombyx mori L*. using a *piggyBac* transposon-derived vector. *Nat. Biotechnol.* **18**, 81–84 (2000).

20. Zhao, A. *et al.* New and highly efficient expression systems for expressing selectively foreign protein in the silk glands of transgenic silkworm. *Transgenic Res.* **19**, 29–44 (2010).

26. Long, D. P. *et al.* FLP recombinase-mediated site-specific recombination in silkworm, *Bombyx mori*. *PLoS ONE* **7**, e40150 (2012).

77. Horn, C. & Wimmer, E. A. A versatile vector set for animal transgenesis. *Dev. Genes Evol.* **210**, 630–637 (2000).

78. Siegal, M. L. & Hartl, D. L. Transgene coplacement and high efficiency site-specific recombination with the Cre/*loxP* system in Drosophila. *Genetics* **144**, 715–726 (1996).
